# Promoter mutagenesis and a massively parallel reporter screen of the *MAPT* locus identifies cis-regulatory elements and genetic variation effects

**DOI:** 10.64898/2026.03.06.710116

**Published:** 2026-03-09

**Authors:** Rebecca M. Hauser, Henry L. Limbo, J. Nicholas Brazell, Belle A. Moyers, Shelby N. Lauzon, Erin A. Barinaga, S. Quinn Johnston, Brianne B. Rogers, Jared W. Taylor, J. Nicholas Cochran

**Affiliations:** 1 HudsonAlpha Institute for Biotechnology, 601 Genome Way, Huntsville, AL, USA 35806

**Keywords:** Tau, MAPT, MPRA, Genetic Variation, Alzheimer’s Disease, Frontotemporal Dementia, Progressive supranuclear palsy, Cis-Regulatory Elements, Dementia, Neurodegeneration, Gene Regulation, Saturation Mutagenesis

## Abstract

Tau neurofibrillary tangles are a hallmark of several neurodegenerative diseases called tauopathies, including frontotemporal dementia and Alzheimer’s Disease. Ongoing clinical trials for tauopathies seek to reduce Tau in the brain through immunotherapy, antisense oligonucleotides, and siRNA. *MAPT* codes for Tau, therefore understanding how the *MAPT* gene is regulated and the effect of genetic variation at its regulatory elements is likely to have high relevance for tauopathies. We screened a ~3 Mb region including the *MAPT* locus using 2 different massively parallel reporter assay (MPRA) strategies in KOLF2.1J h-NGN2 neurons and HEK293FT cells, identifying previously unannotated cis-regulatory elements (CREs). Using CRISPR interference (CRISPRi) in mixed neuron cultures, we identified a new CRE for *MAPT*, as well as 2 CREs for another nearby gene of interest, *KANSL1*. Known genetic variation from the Alzheimer’s Disease sequencing project was tested in a separate MPRA at the top CREs near the *MAPT* gene, identifying variants with altered regulatory effects including those at previously identified CREs for *MAPT*. Using a saturation mutagenesis screen of a 2,000 bp region encompassing the *MAPT* promoter, we assessed regulatory effects of each possible single nucleotide variant in this region. We identified several neuron-specific regulatory variant effects at this region, including a high confidence binding site for the transcription factors EGR2, ZBTB14, and TCLF5 at a region of high MPRA activity and genetic conservation.

## Introduction

Tau is a microtubule associated protein encoded by the gene *MAPT* whose dysregulation plays an important role in several neurodegenerative disorders called tauopathies, including progressive supranuclear palsy (PSP), frontotemporal dementia (FTD), and Alzheimer’s Disease (AD). Alzheimer’s disease is the most common tauopathy, and is considered a secondary tauopathy with the primary pathology being amyloid beta plaques.^1^ In AD, Tau becomes hyperphosphorylated leading to the formation of aggregates called neurofibrillary tangles that precede cell death.^2^ Over time, the burden of Tau aggregates and amyloid plaques increases and correlates with neurodegeneration and the impairment of cognitive ability.

Recently, reduction of Tau has gained interest as a potential target for tauopathy therapeutics. Tau reduction has been shown to have a beneficial effect in animal models of AD without severe deficits.^3–8^ Several pharmaceutical companies have tested immunotherapies targeting Tau with the goal of clearing the pathological protein from the brain.^9–20^ Another strategy being explored to reduce total Tau is targeting *MAPT* with antisense oligonucleotide or siRNA therapy to reduce overall gene expression level.^21–23^ To date, 15 therapies targeting Tau pathology are in development.^24,25^ Due to its shared pathology across multiple diseases and advances in technology allowing us to develop more targeted reduction strategies, it has become increasingly important to understand how *MAPT* is regulated.

The *MAPT* gene is located on chromosome 17 within a well known chromosomal inversion region at 17q21.31. This inversion is sorted into two primary haplotypes H1 (forward orientation) and H2 (inverted orientation). The H1 haplotype is associated with an increased risk for neurodegenerative disorders including Alzheimer’s disease, while the H2 haplotype is considered to have a protective effect in AD.^26^ The primary *MAPT* promoter is GC rich without a TATA box domain, and is located upstream of the first exon of the *MAPT* gene encoding for the 5’UTR.^27,28^ Tau has several different isoforms with unique properties. Alternate splicing of exon 10 results in either 3R or 4R Tau named for the number of repeat domains in the microtubule binding region.^1^ Different tauopathies have varying ratios of 3R and 4R Tau with Alzheimer’s disease typically having a ratio of 1:1 3R to 4R Tau, and progressive supranuclear palsy showing primarily 4R Tau pathology.^29^ In addition, inclusion of exon 4a results in an isoform known as “big Tau.”^30^ Big Tau is primarily located in peripheral neurons^31–33^, and it is possible that its larger size could promote axonal transport. Furthermore, this isoform might be protective against forming aggregates due to a reduction in phosphorylation sites.^34–38^

Recently, we identified several cis-regulatory elements (CREs) for *MAPT.*^39^ We sought to expand our initial study to further understand *MAPT* regulation by performing an unbiased screen of the *MAPT* locus to identify regulatory elements using massively parallel reporter assays (MPRAs). Additionally, we evaluated the effects of genetic variation at the *MAPT* promoter through saturation mutagenesis of a 2,000 bp region encompassing the promoter and evaluated known genetic variation from the Alzheimer’s Disease Sequencing Project (ADSP) database at validated and proximal CREs for *MAPT*. This work increases our understanding of Tau regulation and adds to the growing resource of functional regulatory element screens in neurons.^40–48^

## Results

### MPRA screens identify new cell-type specific CREs

**Figure 1:**
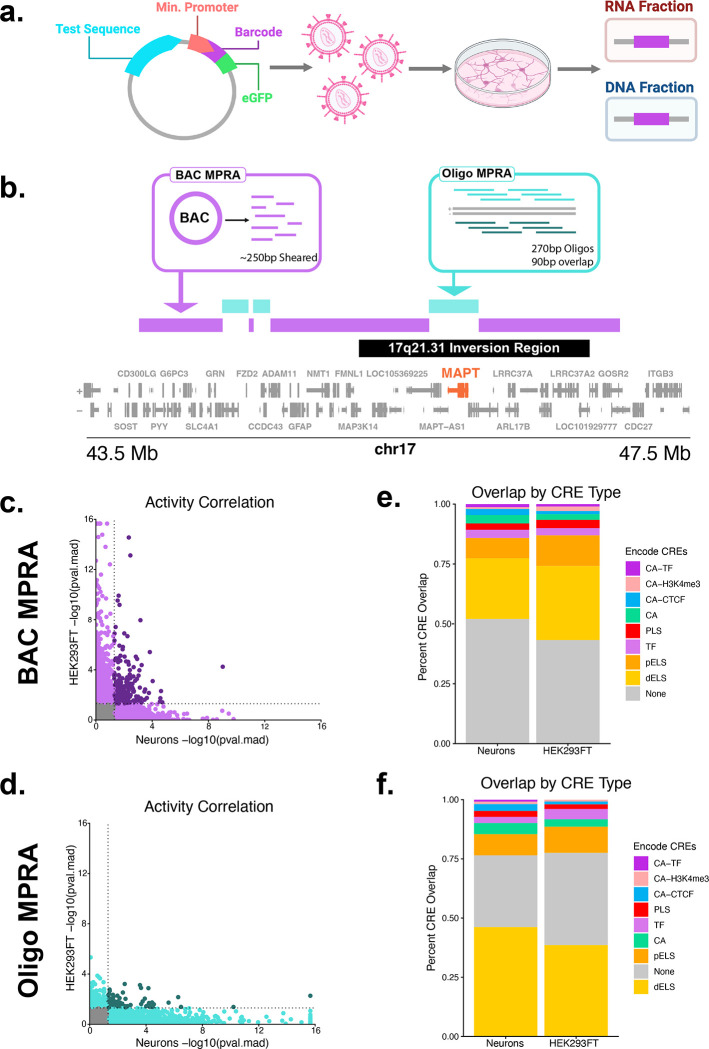
**a**. LentiMPRA experimental design diagram. **b**. Gene track of the MAPT locus and regions of the genome covered by the BAC and Oligo MPRAs. Gaps in the BAC MPRA represent regions of low MPRA coverage where the data was unusable. The Oligo MPRA was designed to supplement these gaps. **c**. Correlation of regulatory activity of 100 bp genomic bins from the BAC MPRA in Neurons and HEK293FT cells. (mad.score Spearman’s ⍴ = 0.0162, approximate p = 0.0395). Pval.mad was capped at a lower limit of 2.2×10^-16^. **d**. Correlation of regulatory activity of 270 bp oligos from the Oligo MPRA in Neurons and HEK293FT cells. (mad.score Spearman’s ⍴ = −0.0464, approximate p = 2.86×10^−4^). **e**. Overlap of ENCODE v4 cCREs with regions identified as regulatory elements through the BAC MPRA (pval.mad ≤ 0.05). Pval.mad was capped at a lower limit of 2.2×10^-16^. **f**. Overlap of ENCODE v4 cCREs with regions identified as regulatory elements through the Oligo MPRA (pval.mad ≤ 0.05). CA-TF = chromatin accessibility with transcription factor binding, CA-H3K4me3 = chromatin accessibility with H3K4me3, CA-CTF = chromatin accessibility with CTCF, CA = chromatin accessibility, PLS = promoter-like sequence, TF = transcription factor binding, pELS = proximal enhancer-like sequence, dELS = distal enhancer-like sequence.

To screen the *MAPT* locus for cis-regulatory elements, we utilized two massively parallel reporter assay strategies. For both MPRAs, the lentiMPRA plasmid was transfected into HEK293FT cells (HEKs) to generate lentivirus to transduce 14 day old KOLF 2.1j-hNGN2 neurons (fig. 1a.).^49,50^ Bacterial artificial chromosomes (BACs) aligning to a ~3 Mb region encompassing the *MAPT* locus were selected and randomly sheared to an average size of 250 bp to use as input for an MPRA (BAC MPRA). A synthesized oligo pool strategy (Oligo MPRA) was implemented for regions of low coverage from the BAC MPRA, including a region directly covering the *MAPT* gene as well as for other regions of interest. 270 bp oligos were designed every 90 bp tiling the forward and reverse strands of genomic DNA in this region and ordered from Twist Biosciences (fig. 1b). For both MPRAs, we observed that the majority of CREs were specific to each tested cell type. We identified 1,636 bins with positive regulatory activity in neurons and 2,257 in HEKs with an overlap of 266 shared bins per cell type in the BAC MPRA (pval.mad ≤ 0.05) (fig. 1c, tables s2-3). We identified 2,900 oligos with significant positive regulatory activity in neurons and 328 oligos in HEKs with an overlap of 84 shared between the two cell types in the Oligo MPRA (pval.mad ≤ 0.05) (fig. 1d, tables s4-5). When overlapping and consecutive significant bins/oligos were joined to form a single CRE, we identified 1,335 CREs using the BAC MPRA in neurons and 1,402 CREs in the HEKs as well as 864 CREs in the Oligo MPRA in neurons and 254 in HEKs (fig. s1). Comparing our MPRA data to annotated ENCODE v4 cCREs^51^, the majority of CREs found using the BAC MPRA were previously unannotated (fig. 1e), and the majority of the CREs found using the Oligo MPRA were annotated as enhancer-like sequences (dELS) (fig. 1f).

### CRISPRi identifies new CREs for MAPT and KANSL1

**Figure 2:**
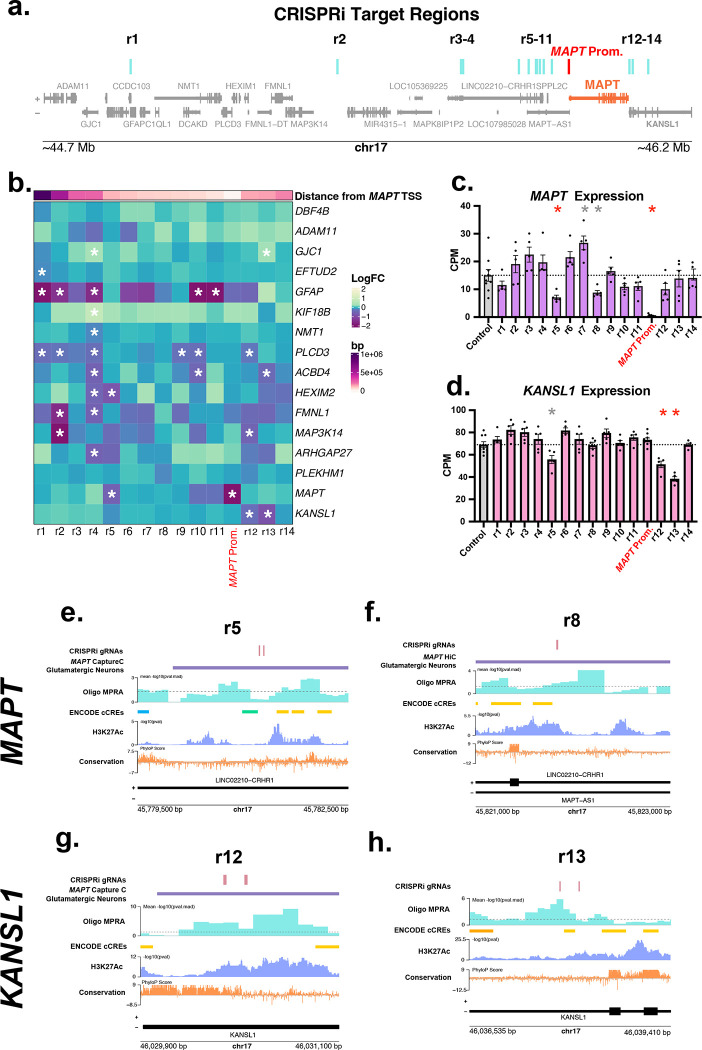
**a**. Gene track showing the locations of the nominated CRISPRi target regions in relation to the MAPT gene. Blue lines indicate target regions, and the red line is the location of the guide for the MAPT promoter positive control. **b**. Heatmap of genes 200kb away from a CRIPRi target region normalized for cell type score. Asterisks represent an adj. p. value ≤ 0.05. **c**. Barplot of MAPT CPM when targeting each nominated region with CRISPRi. Red asterisks represent targets with an adj. p. value ≤ 0.05 from DESeq2 both before and after normalizing for cell type score, gray asterisks represent an adj. P. value ≤ 0.05 from DESeq2 without normalizing for cell type score. **d**. Barplot of KANSL1 CPM when targeting each nominated region with CRISPRi. Red asterisks represent targets with an adj. p. value ≤ 0.05 from DESeq2 both before and after normalizing for cell type score, gray asterisks represent targets with an adj. P. value ≤ 0.05 without normalizing for cell type score. **e**–**h**. Gene tracks of region r5, r8, r12, and r13 respectively. The dotted line on the MPRA signal track represents a pval.mad of 0.05.

From the CREs identified in the initial MPRA screens, we nominated 14 regions as potential regulatory elements for *MAPT* by considering overlap of previously published 3D -omics data^39^ and multiomics data^39,52^ and incorporating both the distance from the *MAPT* promoter and the strength of the enhancer activity in our MPRAs (fig. 2a).^39,52^ CRISPRi guides were designed to each region and were transduced into KOLF 2.1J NPCs.^39^ NPCs were differentiated following the the Bardy et al. protocol,^53^ which produces a mixed culture of inhibitory and excitatory neurons, as well as some immature astrocytes. RNA was collected from the cells and sent for mRNA-sequencing. Because *MAPT* is expressed in both neurons and astrocytes, to account for the multiple cell types present in the bulk RNA as well as well-to-well cell count variability, Seurat^54^ cell type scores for neurons and astrocytes were added to the DESeq2^55^ model to ensure that changes in *MAPT* expression were due to *MAPT* repression and not a reduction in total neuron count (table s9). Expression of genes within 200 kb of the guides were examined to look for likely gene targets by proximity of the candidate enhancers (fig. 2b).^56^ We discovered a novel enhancer for *MAPT* at region r5 (adj. p. value = 2.24×10^−4^ and 0.0213 before and after accounting for cell type score) ~113,349 bp upstream of the *MAPT* TSS falling within a *MAPT* promoter Capture-C link identified in cultured neurons.^39^ Excluding the cell type score from the DESeq2 model, repression of region r8 located ~72,708 bp upstream of the *MAPT* TSS within a *MAPT* promoter HiC link also resulted in a significant reduction of *MAPT* expression (adj. p. value = 0.0145 and 0.990 before and after accounting for cell type score). This is likely due to an overall reduction of neuron count caused by a prevention of differentiation of NPCs into neurons following the blocking of this CRE with CRISPRi. We are reporting this region as a CRE important for neuronal differentiation and can not exclude that it is acting directly on *MAPT* regulation (fig. 2c). In the process of testing these CREs, we found regions r12 and r13 to be CREs for the gene *KANSL1* (r12 adj. p. values = 4.86×10^−6^, 2.94×10^−6^ before and after adjusting for cell type score, and r13 adj. p. values < 2.2×10^−16^ both before and after adjusting for cell type score). Pathogenic variants within *KANSL1* are known to cause the developmental disorder Koolen-de Vries Syndrome.^57^ In addition, variants at the *KANSL1* locus in connection with H1/H2 haplotypes are associated through genome-wide association studies with increased AD, Parkinson’s disease, and amyotrophic lateral sclerosis risk.^58,59^ Region r5, in addition to being a high confidence CRE for *MAPT* also decreased *KANSL1* expression when targeted by CRISPRi, although this association was lost once accounting for cell type score (fig. 2d) (adj. p. value = 2.03 × 10^−3^ and 0.118 before and after adjusting for cell type score). Several CREs affected expression of the *GFAP* gene. We believe that region r1 (adj. p. value = 3.68×10^−12^ and 1.07×10^−16^ before and after adjusting for cell type score) could be a CRE for GFAP due to its proximity to the *GFAP* promoter (between 2 ENCODE CREs within *GFAP* exon 9^60^), however we cannot be certain if the other CREs are directly influencing *GFAP* expression or indirectly affecting it through other mechanisms including altering astrocyte reactivity.^61^

### MPRAs identify regulatory variant effects at proximal CREs to MAPT

**Figure 3:**
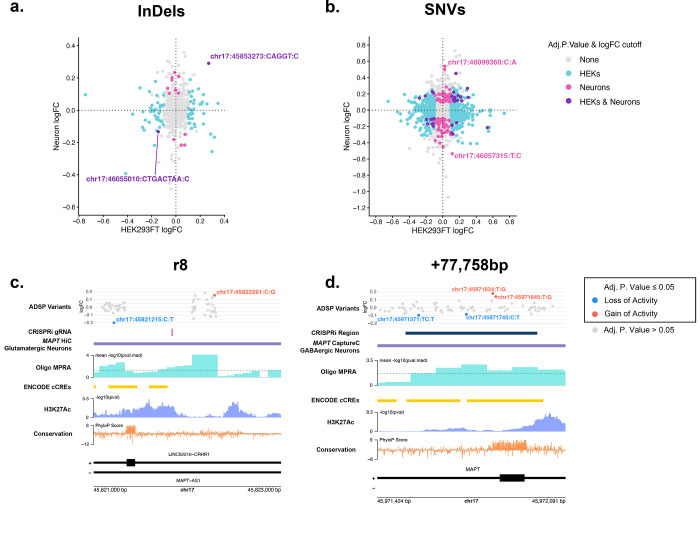
**a**–**b**. Correlation plots of (a) InDels (logFC Spearman’s ⍴ = 0.0649, approximate p = 0.114) and (b) SNVs (logFC Spearman’s ⍴ = 0.0865, approximate p = 1.52×10^−15^) variant effects from the ADSP database (both Alzheimer’s cases and unaffected controls). Blue, pink, and purple points represent variants with an adj. p. value ≤ 0.05 as well as a |logFC| > 0.1 in HEK293FT, Neurons, and both cell types respectively. Gray points represent variants that either have an adj. Pp value > 0.05 or a |logFC| < 0.1 **c**–**d**. Gene tracks showing (c) region r8 and (d) a CRE for MAPT identified by Rogers et al.^39^ respectively. The dotted line on the MPRA signal represents a pval.mad of 0.05. Variant effects are shown as logFC in neurons.

To determine the effects of genetic variation at CREs surrounding *MAPT*, the top 25% of CREs from the Oligo MPRA surrounding *MAPT* in neurons, as well as CREs previously identified for *MAPT*^39^ were selected as input into a variant screening MPRA. From the Alzheimer’s Disease Sequencing Project (ADSP) database, we selected single nucleotide variants (SNVs) and insertions/deletions (InDels) that overlapped these regulatory regions and were present in both Alzheimer’s Disease and non-Alzheimer’s Disease groups. We chose to exclude InDels greater than 10 bp. The MPRA was transfected into HEK293FT cells to generate lentivirus to transduce 14 day old KOLF2.1-hNGN2 neurons. DNA and RNA were collected from both cell types, and variant effects were quantified by bcalm^62^, with a |logFC| cut-off of 0.1 by comparing to reference sequence controls. The majority of variant effects were cell-type specific, and 2 InDel effects were shared between cell types (the deletions chr17:45853273:CAGGT:C (within *MAPT-AS1* intron 1), and chr17:46055010:CTGACTAA:C (within *KANSL1* intron 6)) (fig. 3a, tables s10-11). We identified 117 InDels showing a loss of activity in HEKs and 37 showing a gain of activity, as well as 5 showing a loss of activity and 9 showing a gain of activity in neurons (adj. p. value ≤ 0.05 and |logFC| > 0.1). SNVs showed a similar pattern with the majority of variant effects being cell-type specific (fig. 3b, tables s12-13). 55 SNVs showed an increase in activity and 61 a decrease in activity in neurons (adj. p. .value ≤ 0.05 and |logFC| > 0.1). 447 and 433 SNVs showed a loss and gain of activity respectively, in HEKs, and 9 SNVs showed a loss of activity in both HEKs and neurons with 13 showing a gain of activity in both cell types. The SNVs with the largest effect sizes in neurons were chr17:46099360:C:A (within *KANSL1* intron 2) which increased activity of the CRE (adj. p. value = 0.0269), and chr17:46057315:T:C (within *KANSL1* intron 6) that showed a loss of activity (adj. p. value = 0.0246). Since the variants tested were chosen within regions that were CREs identified in neurons and not necessarily CREs in HEKs, that could contribute additional noise to the HEK variant effects. The candidate *MAPT* CRE region r8 showed a variant with a loss of activity at chr17:45821215:C:T in neurons (adj. p. value = 3.76×10^−5^ ) and gain of activity at chr17:45822291:C:G (adj. p. value = 0.0284) (fig. 3c). A CRE identified by Rogers et. al. at +77,758 bp from the *MAPT* promoter^39^ had 4 variant effects with chr17:45971571:TC:T and chr17:45971740:C:T showing a loss of activity and chr17:45971834:T:G and chr17:45971845:T:G showing a gain of activity in neurons (adj. p. values = 0.0221, 0.0495, 1.17×10^−5^, and 5.42×10^−3^ respectively) (fig. 3d). It should also be noted that this region also overlapped with regulatory regions in Cooper et al.^42^ and Corces et al.^63^

### Saturation mutagenesis comprehensively evaluates SNV regulatory effects at the MAPT promoter

**Figure 4:**
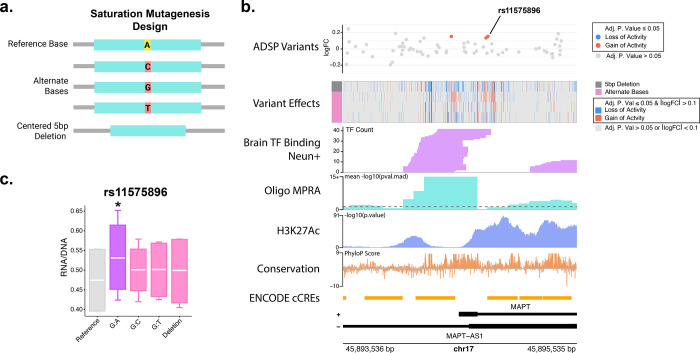
**a**. Graphical overview of MAPT promoter saturation mutagenesis design. **b**. Gene track for the MAPT promoter saturation mutagenesis region. The top track is variants found in ADSP in this region. Red points represent variants with an adj. p. val ≤ 0.05 with an increase of regulatory activity relative to the reference sequence. The second track is a heatmap showing variants that had an adj. p. val ≤ 0.05 as well as a logFC in activity of > 0.1 in red and < 0.1 in blue. Red lines indicate variants with an increase in regulatory activity, and blue lines indicate variants with a decrease in regulatory activity. The top row of the heatmap is data from the 5 bp deletions, the subsequent 3 rows are data from the 3 alternate bases at that position. The third track shows transcription factor binding in neurons, data from Loupe et al.^64^ The dotted line on the MPRA signal track indicates a pval.mad of 0.05. **c**. Box plot showing RNA/DNA ratio for rs11575896, the reference sequence, each alternate base tested, and 5 bp deletion at this region. Whiskers show maximum and minimum values. The asterisk indicates an adj. p. value ≤ 0.05 by bcalm.

To comprehensively assess the effect of genetic variation in the *MAPT* promoter, a 2,000 bp region encompassing the promoter was selected, and an MPRA was designed with 219 bp oligos centered on every base in this region (chr17:45,893,536–45,895,535). Oligos were designed with each possible alternate base relative to the reference sequence. To completely ablate any transcription factor binding sites, a centered 5 bp deletion sequence was also designed (fig. 4a). Activity signal from the initial Oligo MPRA in this region aligns with transcription factor binding sites in neurons (fig. 4b).^64^ This HOT (high occupancy target)^65,66^ site (chr17:45,893,810–45,894,854) was also enriched for single nucleotide regulatory variant effects with 332 of the 391 SNVs variant effects identified falling in this region (fig. 4b). Several variants in this region were present in the ADSP database, of note the common SNV rs11575896 (allele frequency = 0.1436, gnomAD v4.1.0^67^) shows an increase in activity that is specific to the G:A substitution (adj. p. value = 1.19×10^−3^) (fig. 4c). In addition, chr17:45894238:C:G showed an increase in enhancer activity in our study in neurons (logFC = 0.141, adj. p. value = 8.68×10^−3^) as well as in neural progenitor cells by Gaynor-Gillett et al.^43^ However, this variant had the opposite effect in HEKs (logFC = −0.0569, adj. p. value = 0.0414). Overall, we had 192 SNVs showing a loss of regulatory activity (adj. p. value ≤ 0.05 and logFC < −0.1), and 199 showing a gain of regulatory activity (adj. p. value ≤ 0.05 and logFC > 0.1). 142 and 82 centered 5 bp deletions had a loss and gain of activity, respectively (adj. p. value ≤ 0.05 and |logFC| > 0.1) (fig. 4b, table s14). In HEK293FT cells, we observed 523 SNVs and 417 SNVs with a gain and loss of activity, respectively, as well as 226 and 229 centered 5 bp deletions with a gain or loss of activity, respectively (adj. p. value ≤ 0.05 and |logFC| > 0.1) (fig. s3a, table s15).

### Correlation of AI predictive tools and MPRA results identify a high confidence loss of activity region in the MAPT promoter

**Figure 5:**
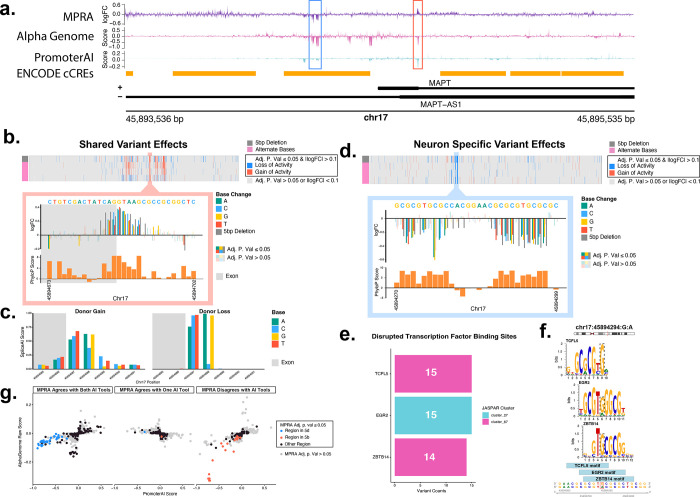
**a**. Gene track showing logFC of each SNV tested in the MAPT promoter region (top), AlphaGenome score for each variant in this region in glutamatergic neurons (middle), and PromoterAI (bottom) score for each variant in this region. The blue box represents the region in 5d and the red box represents the region in 5b **b**. (top) Heatmap showing variants with an adj. P. value ≤ 0.05 by bcalm and a |logFC| > 0.1 that are shared between neurons and HEK293FT cells. The top row of the heatmap shows data from 5 bp centered deletions at that position, the bottom 3 rows show data from alternate bases at that position. Variants showing an increase in regulatory activity compared to reference are shown in red and variants with a decrease in regulatory activity are shown in blue. (bottom) logFC of regulatory activity of each variant in the highlighted region compared to the reference sequence. Opaque bars have an adj. P. value ≤ 0.05 by bcalm and transparent bars have an adj. P. value > 0.05. Bars with a gray background are in exon 1 of MAPT and bars with a white background are in intron 1 of MAPT. Reference sequence is shown on top of the graph. Below the top graph is a bar plot of PhyloP genetic conservation score^71,72^ for each base. **c**. Bar plots showing SpliceAI scores for each alternate base in this region. Bars with a gray background are in exon 1 of MAPT and bars with a white background are in intron 1 of MAPT. **d**. (top) Heatmap showing variants with an adj. P. value ≤ 0.05 by bcalm and a |logFC| > 0.1 that are present in neurons but not in HEK293FT cells. The top row of the heatmap shows data from 5 bp centered deletions at that position, the bottom 3 rows show data from alternate bases at that position. Variants showing an increase in regulatory activity compared to reference are shown in red and variants with a decrease in regulatory activity are shown in blue. (bottom) logFC of regulatory activity of each variant in the highlighted region compared to the reference sequence. Opaque bars have an adj. P. value ≤ 0.05 by bcalm and transparent bars have an adj. P. value > 0.05. Bars with a gray background are in exon 1 of MAPT and bars with a white background are in intron 1 of MAPT. Reference sequence is shown on top of the graph. Below the top graph is a bar plot of PhyloP genetic conservation score^71,72^ for each base. **e**. Transcription factor binding sites that are disrupted by variants with a significant loss of activity in the highlighted region in d. in motifbreakR colored by JASPAR motif clusters. **f**. Precision weight matrices of disrupted transcription factor binding sites in the highlighted region in d. Pictured is the base chr:45894294:G:A which is one of the variants with a significant loss of activity in the highlighted region in d. **g**. Correlation plots of AlphaGenome and PromoterAI scores separated by whether the direction of the predicted variant effect agrees with the direction of the logFC seen in the MPRA. Gray dots indicate an adj. p. value > 0.05 by bcalm. Black points indicated an adj. P. value ≤ 0.05 not within a highlighted region. Blue points indicate regions with an adj. P. value ≤ 0.05 in the highlighted region in **d**. Red points indicate regions with an adj. P. value ≤ 0.05 in the highlighted region in b.

Recently, there has been an increase in the use of AI tools in the interpretation of genomic datasets. We input the SNVs introduced in the saturation mutagenesis of the *MAPT* promoter into the AI tools AlphaGenome’s saturation mutagenesis function^68^and PromoterAI^69^, and compared the predicted regulatory effects on the *MAPT* gene with the activity of variant effects in our MPRA (tables s16-17). Overall, there was correlation between scores from AlphaGenome and PromoterAI (Spearmans’s ⍴ = 0.153, approximate p < 2.2×10^−16^) (fig. s4, fig. 5a). We compared the results of the saturation mutagenesis in both HEKs and neurons to determine variant effects specific to the neuron cell type, as well as variant effects shared between both cell types. Of the SNVs effects found in our *MAPT* promoter mutagenesis, 195 variant effects were shared between cell types (adj. p. value ≤ 0.05, |logFC| > 0.1 or < −0.1, with shared directionality of logFC in both cell types) and 186 were specific to neurons (fig. s3b, fig. 4b). Within the shared variant effects, there was a region at chr17:45,894,685–45,894,691 where any base substitution resulted in a gain of regulatory activity as measured by our MPRA (fig. 5b); however, this region shows a strong predicted loss of *MAPT* gene expression by both AlphaGenome and PromoterAI (fig. 5a). This region overlaps the *MAPT* exon1-intron1 boundary, and the variants are predicted to result in alternate splicing of the *MAPT* gene by SpliceAI^70^ (fig. 5c), accounting for the predicted loss of *MAPT* expression by the AI tools despite the increase in activity seen in the MPRA output. This underlies the importance of considering genomic context when interpreting results from MPRAs incorporating genetic variants. Among the neuron-specific variant effects, we observed there to be a region around chr17:45,894,271–45,894,297 where any base substitution resulted in a loss of regulatory activity (fig. 5d). This region overlaps an area of strong activity signal from our initial MPRA as well as an area of high transcription factor binding (fig. 4b). The peaks of loss of activity correspond to peaks of high genetic conservation scores^71,72^, and we believe this to be a critical transcription factor binding site for *MAPT* expression in neurons. MotifbreakR^73^ was used to determine what transcription factor binding sites were disrupted by the variants at these positions. We saw that in this region binding sites were disrupted for the transcription factors TCFL5, EGR2, and ZBTB14 (fig. 5e–f, table s20). When comparing the scores given by AlphaGenome and PromoterAI with the results from the MPRA, we saw that the neuron-specific loss of activity region (in fig. 5d) was the region of highest agreement among all three tools, while the exon1-intron1 boundary region (in fig. 5b) was the area of highest disagreement among the MPRA results and AI tools (fig. 5g).

## Discussion

We screened a ~3 Mb region around the *MAPT* locus for cis-regulatory elements in human iPSC derived excitatory neurons using lentiMPRAs. The results of these screens identified several previously unannotated regulatory elements and also validated activity of existing ENCODE cCREs. Our results show the importance of testing CREs in cell types of interest, as the majority of regulatory elements we identified were cell-type specific. We believe this also accounts for the findings of multiple new previously unannotated CREs in this region. Using known genetic variation from Alzheimer’s disease patients and non-Alzheimer’s disease affected individuals from the ADSP database, we were able to identify variants that altered the activity of the CREs with the highest activity around *MAPT.* These variants likewise showed strong cell type specificity. We identified regulatory variant effects at CREs for *MAPT* identified in Rogers et al. at regions −674,458; −652,338; −464,677; −461,949; −44,905; −20,178; and +77,758 bp away from the *MAPT* promoter.^39^ Due to their location within validated CREs for *MAPT*, these variants have the potential to alter *MAPT* expression although their direct effect on *MAPT* was not tested in this study.

We comprehensively mapped genetic variant effects at a 2,000 bp region encompassing the *MAPT* promoter (chr17:45,893,536–45,895,535) and identified 199 variants with an increase in regulatory activity and 192 variants with a decrease of regulatory activity relative to the reference genome in neurons. Of note, we identified 145 neuron-specific single nucleotide regulatory variants that were located in a HOT site region of the *MAPT* promoter (chr17:45,893,810–45,894,854) shown by strong MPRA activity and transcription factor binding in neurons that have an increased likelihood to alter expression of *MAPT*, including a variant also found by Gaynor-Gillett et al. in neural progenitor cells.^43^ We also identified a ~26 bp region at chr17:45,894,271–45,894,297 with a high confidence reduction in regulatory activity (fig. 5d). Using motifbreakR, we saw that this region was enriched for the binding motifs of the transcription factors EGR2, TCLF5, and ZBTB14. EGR2 is an immediate early gene (IEG) heavily implicated in promoting myelination^74–78^ and Charcot-Marie Tooth Disease^79–89^ as well as being implicated in memory, epilepsy, inflammatory response, and Rett syndrome.^82–89^ Interestingly, *EGR2* was recently shown to be upregulated in the hippocampus of a presymptomatic rat model of AD, and *EGR2* deficient mice show an improvement in learning and memory.^82,90^ ZBTB14 is implicated in seizure regulation^91^, and TCLF5 has been studied in spermatogenesis.^92,93^ It is worth noting that both EGR2 and ZBTB14 were targeted in a genome wide CRISPRi screen in human iPSC-derived neurons by Samelson et. al. where no effect was observed on levels of Tau oligomers.^94^ Likewise, EGR2 was tested in shRNA screens in human Daoy cells as well as in drosophila and with no change in Tau protein levels.^95^ Since neither of these papers measured gene expression, it is possible that these transcription factors modulate *MAPT* gene expression in a way that is not translated to protein expression levels. The transcription factors could also be acting in a cell-type and species specific way, and since we did not directly observe the effect of these transcription factors on *MAPT* expression it is possible that other transcription factors with similar binding motifs are responsible for the loss of activity at this region observed in our MPRA. In addition, this is not the only region of the *MAPT* promoter responsible for inducing *MAPT* transcription. The *MAPT* promoter itself is a genomic HOT site with multiple transcription factors binding this region (fig. 4b), including CLOCK, EGR1, and NRF1 (table s18).^64^

With the advent of new AI tools to predict the regulatory effects of genetic variation, we compared the results from our saturation mutagenesis MPRA with two AI tools, PromoterAI and AlphaGenome. Overall, there was a correlation between two tools (Spearmans’s ⍴ = 0.153, approximate p < 2.2×10^−16^). However, PromoterAI had a higher correlation with the results from our MPRA (Spearmans’s ⍴ = 0.482, approximate p = 1.89×10^−4^ PromoterAI, and Spearmans’s ⍴ = 0.0277, approximate p = 0.0321 AlphaGenome). We observed that the region with the highest agreement among all three tools was also the region of largest variant effects measured by our MPRA. It is worth noting that the MPRAs do not reflect direct regulation of the *MAPT* gene, only accounting for isolated increases or decreases in regulatory activity of the tested sequences in each cell type. The AI tools strive to predict expression effects of specific genes by pulling in multiple predictive datasets, accounting for some of the differences between our MPRA and the AI tools. We believe that the field is not yet at a point where MPRAs can be excluded altogether in favor of AI predictive tools due to the highly cell type specific nature of gene regulation, but they can aid in the interpretation of MPRA datasets as we saw with the splice variant region that was in high agreement between both AI tools but showed an increase in activity in our saturation mutagenesis MPRA.

Limitations of this study include that all experiments were done in cell types containing the H1 haplotype, and it is possible that experimental results would be different in H2 containing cells. In addition, the direct effect of genetic variation on *MAPT* expression was not evaluated at this time, and is beyond the scope of this study (requiring techniques such as prime editing, which could be the focus of future studies). Additionally, the only cell types used in this study were excitatory neurons from the KOLF2.1Jh-NGN2 line, HEK293FT cells, or mixed KOLF2.1J neuron cultures and other cell types were not tested. Despite these limitations, this study makes strong advances in understanding the effects of regulatory elements and related genetic variation on expression of *MAPT* and other genes near *MAPT* also affected by the H1/H2 inversion event associated with several neurodegenerative diseases.

## Methods

### BAC MPRA

#### Library Design

24 BACs clones (fig. s1, table s1) were selected from BacPac Resources (https://bacpacresources.org) that aligned to a region approximately 3 Mb around the *MAPT* locus. BACs were grown in 5mL Terrific Broth (Corning 46–055-CM) with 12.5μg/mL (sigma C0378–5G). 5μg of BAC DNA was purified using QIAGEN plasmid buffer set (QIAGEN 19046) and QIAGEN-tip 500 (QIAGEN 10063), and sheared to an average size of around 250 bp using a Covaris S220. Sheared BACs were prepped for cloning as previously described^96^, and they were inserted into pLS-SceI following the published protocol.^97^ Following cloning and electroporation into NEB 10-beta electrocompetent cells (New England Biolabs C3020K), a 500mL LB broth (Corning 46–050-CM) + 100μg/mL carbenicillin (Sigma C1389) culture was grown at 37°C and 150rpm for 13 hours. The plasmid pool was purified using the QIAGEN plasmid plus mega kit (QIAGEN 12981). A subset of the culture was spread on LB agar plates (Millipore 1102830500) with 100μg/mL carbenicillin (Sigma C1389) at 37°C, and several colonies were chosen for sequence validation with Sanger Sequencing before sending the pool for Barcode Association Sequencing. pLS-SceI was a gift from Nadav Ahituv (Addgene plasmid # 137725; http://n2t.net/addgene:137725; RRID:Addgene_137725)^97^.

#### Barcode Association

The locus was divided into 100 bp nonoverlapping bins. The sheared BAC sequences were aligned to HG38, and a custom script was used to aid in the association of each 15 bp barcode with a 100 bp bin based off of which bin the middle of the associated sheared BAC sequence was aligned. Libraries were prepared according to Gordon et al^97^ and sequenced on an Illumina NextSeq flowcell.

### Oligo MPRA

#### Library Design

270 bp oligos were ordered from Twist biosciences every 90 bp for the forward and reverse DNA sequences of regions of interest (ROI). Total, this gives 6X coverage of each ROI tested in each cell type. For analysis, forward and reverse strands were combined into 1 ROI. Regions were chosen based on areas with low coverage in the BAC MPRA, tiling the *MAPT* locus (Hg38 chr17:45,771,561–46,100,061), as well as further upstream (Hg38 chr17:44,400,964–44,575,474 and Hg38 chr17:44,605,938–44,718,798). Several regions were included for other genes of interest based on multiomics data from previous publications (Anderson and Rogers et al.^52^ and Cochran et al.^98^), and 100 scrambled sequences were added as negative controls.

The oligo pool was inserted into pLS-SceI according to the previously published protocol.^97^ Following cloning and electroporation into NEB 10-beta electrocompetent cells (New England Biolabs C3020K), a 500mL LB broth (Corning 46–050-CM) + 100μg/mL carbenicillin (Sigma C1389) culture was grown at 37°C and 150rpm for 13 hours. The plasmid pool was purified using the QIAGEN plasmid plus mega kit (QIAGEN 12981). A subset of the culture was spread on LB agar plates (Millipore 1102830500) with 100μg/mL carbenicillin (Sigma C1389) at 37°C, and several colonies were chosen for sequence validation with Sanger Sequencing before sending the pool for Barcode Association Sequencing on an Illumina Novaseq.

### Variation MPRA

#### MAPT Promoter Mutagenesis

219 bp oligos were designed for a 2,000 bp region surrounding and including the *MAPT* promoter (Hg38 chr17:45,893,536–45,895,535) and ordered from Twist Biosciences. For every base in this region, an oligo was designed where the reference sequence is centered on the position of interest as well as 3 additional oligos with each possible alternate base at that position and an oligo with a centered 5 bp deletion. The mutagenesis and known variant MPRAs were combined in a single 36,000 Oligo MPRA pool and separated at the time of analysis.

#### Known Variants

SNVs and InDels of no more than 10 bp were chosen from the ADSP^13^ database that overlapped the top 25% of CREs in neurons near *MAPT* (Hg38 chr17:45,771,561–46,100,061) found using the Oligo MPRA, regions chosen for CRISPRi testing from the BAC or Oligo MPRA, the *MAPT* promoter (Hg38 chr17:45,893,536–45,895,535), and regions that were shown as CREs for *MAPT* (+100 bp on each side) from a previous publication (Rogers et al.^39^). For SNVs, 219 bp oligos were designed with the variant centered for both the reference sequence and the SNV. For deletions, the reference seq was 219 bp for deletions of an odd number of bases and 218 bp for deletions of an even number of bases in order to center the deletion region. The variant sequences were the length of 219 or 218 bp minus the number of bases being deleted. For insertions, the variant sequence was 219 or 218 bp (depending on if it was an even or odd number of bases inserted) in order to have the inserted sequence centered within the reference sequence. Reference sequences were 219 or 218 bp long minus the number of bases inserted in the alternate allele. Variants with an allele count of 0 were excluded and variants with an allele frequency of 1 or more were represented 2X. A total of 627 InDels and 9,090 SNVs were included. Since the majority of included regions were previously positive in an MPRA, reference sequences for these regions can act as positive controls.

12 promoters were included as additional positive controls and were represented 2X. The scrambled controls were included from the Oligo MPRA, as well as 2,510 MPRA negative regions and 2,539 GC-matched scrambled negative controls from the tested regions. A subset of negative controls were shortened so that there was a length match to each length of tested regions in the oligo pool, as well as altering the middle base of the scrambled region to each alternate base. The oligo pool was ordered from Twist biosciences. Plasmid libraries were prepared as in Gordon et al.^97^ with a slight modification as the protocol was modified as previously described in order to test both orientations of the insert.^99^ Following cloning into pLS-SceI and electroporation into NEB 10-beta electrocompetent cells (New England Biolabs C3020K), a 500mL LB broth (Corning 46–050-CM) + 100μg/mL carbenicillin (Sigma C1389) culture was grown at 30°C and 150rpm for 20 hours. The plasmid pool was purified using the QIAGEN plasmid plus mega kit (QIAGEN 12981). A subset of the culture was spread on LB agar plates (Millipore 1102830500) with 100μg/mL carbenicillin (Sigma C1389) at 37°C overnight, and several colonies were chosen for sequence validation with Sanger Sequencing before sending the pool for Barcode Association Sequencing on an Illumina NovaSeq X Plus.

### MPRAs

We followed the LentiMPRA protocol.^97^ Briefly, Kolf 2.1J-NGN2 iPSCs were obtained from Jackson laboratory (JIPSC002070). iPSCs were grown as aggregates in matrigel coated flasks in MTESR Plus media (Corning 354277, StemCell Technologies 100–0276). Cells were treated with accutase (Stemcell Technologies 07920) and were plated as single cells onto 15cm plates coated with both poly-l-ornithine (Sigma P4957) and Matrigel (Corning 354277) at a density of 25,000 cells/cm^2^ in induction media (DMEM/F12 with HEPES Gibco 11039021, 1X N2 Supplement-A Stemcell Technologies 07152, 1X Glutamax ThermoFisher 35050061, 1X MEM non-essential amino-acids Gibco 11140050, and at plating 10 μM ROCK inhibitor (Y-27632) (Stemcell technologies 72304)). Doxycycline (Stemcell Technologies 72742) was added to the media at a concentration of 2μg/mL to induce *NGN2* expression and differentiation into excitatory neurons. The cell media was swapped to cortical neuron media (BrainPhys Neuronal Medium Stemcell Technologies 05790, 1X NeuroCult SM1 Neuronal Supplement Stemcell Technologies 05711, 5μg human recombinant BDNF Stemcell Technologies 78005, 5μg human/mouse recombinant NT-3 Stemcell Technologies 78074, 0.5mg Laminin Mouse Protein Gibco 2307015) and allowed to differentiate for 14 days. 1 hr before transduction with lentivirus, the media was swapped to cortical neuron media containing protamine sulfate (Sigma-Aldrich P3369) at a concentration of 10 μg/mL. Lentivirus was made by transfecting HEK293FT cells plated at a density of 73,000 cells/cm^2^ in a T225 flask in supplemented DMEM media (Gibco 11965–084, 10% FBS Cytivia SH30071.03, 1X Glutamax ThermoFisher 35050061, 1X MEM non-essential amino-acids Gibco 11140050, Geneticin 50μg/mL Gibco 10131035). The following day, the media was swapped to Optimem + Glucose (Gibco 31985070, 0.6mg/L Sigma G8644, 1X Glutamax ThermoFisher 35050061). 2 days later the media from 2 T225 flasks per biological MPRA replicate was filtered at 0.45μm (ThermoFisher 09–740-63A). 11.25mL of virus was added to each 15cm plate of neurons. 24hrs following transduction, the media was swapped to fresh cortical neuron media. Cells were collected on day 18 using the Qiagen DNA/RNA kit and on-column DNAse digestion (QIAGEN 80204 and 79254). DNA and RNA from 2 15cm plates were combined for each biological replicate of the MPRAs. Libraries were prepared as in Gordon et al.^97^ and sequenced on a mixture of Illumina NovaSeq X Plus (Variant MPRA, Oligo MPRA, BAC MPRA), Illumina Novaseq (Oligo MPRA), and Illumina NextSeq (BAC MPRA) flowcells. All mammalian cells were grown at 37°C and 5% CO_2_.

#### MPRA Analysis

For the Oligo MPRA, the barcode association dictionary was generated with MPRAflow.^97^ The BAC MPRA and Oligo MPRA both used MPRAflow^97^ to generate DNA and RNA barcode counts. MPRAnalyze^100^ was used to analyze both MPRAs. For the variant and mutagenesis MPRAs, barcode association dictionaries were generated with MPRAflow^97^ without a mapping quality filter. Oligos of different lengths were associated with barcodes separately using a CIGAR score filter requiring a perfect match according to the length (219M, 218M, etc.). The barcode dictionaries were combined, and barcodes associating with multiple oligos were removed. Counts of each barcode in the RNA and DNA fractions from each cell type were generated with MPRAsnakeflow^101^ and input into bcalm.^62^ Known InDels, SNVs, and the promoter mutagenesis were analyzed separately. InDels and SNVs alternate sequences were compared to reference sequence controls. InDels and SNVs were analyzed separately to avoid any effect of altering oligo sizes in the InDels to affect the model for SNV analysis. For the saturation mutagenesis, each alternate base as well as the 5 bp deletion were compared to the reference sequence for that region. Both orientations of test sequences were combined into 1 region for analysis. motifbreakR^73^ was used to identify transcription factor binding sites. SpliceAI^70^ scores were accessed from https://spliceailookup.broadinstitute.org.

#### CRISPRi

gRNAs were designed for each region of interest using Benchling and were ordered from IDT (table s6). The guides were cloned into the pLV hU6-sgRNA hUbC-dCas9-KRAB-T2a-Puro vector^102^ (addgene 71236) using FASTAP (ThermoFisher EF0651) and ESP3I and in 10X fast digest buffer (ThermoScientific FD0454). Lentivirus was created by transfecting HEK293FT (Invitrogen R70007) cells plated at 700,000 cells/well in a PLO (Sigma 00107) coated 6 well plate in supplemented DMEM media (as above) and cells were transfected with 0.75μg psPAX2 (Addgene 12260), 0.5μg pMD2.G (Addgene 12259), and 0.5μg pLV hU6-sgRNA hUbC-dCas9-KRAB-T2a-Puro (Addgene 71236) containing each gRNAs to each target region of interest with Lipofectamine LTX (Invitrogen 15338100). Kolf2.1 NPCs were generated as previously described.^39^ and plated at a density of 200,000 cells per well in a matrigel coated (Corning 354230) 12 well plate in NPC media (350mL DMEM Gibco 11995081, 150mL Ham’s F12 Gibco 11765054, 1X B-27 Supplement Gibco 17504044, 40ng/mL bFGF R&D Systems 233-FB, 20ng/mL hEGF Sigma E9644, heparin 5μg/mL Sigma H3149). The following day, media from the HEK293FT cells was filtered using a 0.45μm PES syringe filter (VWR 76479–020) to obtain CRISPRi packaged lentivirus. Protamine sulfate 10μg/mL (Sigma-Aldrich P3369) was added to the NPC media at a concentration of 10 μg/mL 1 hour before transduction, and NPCs were transduced with 500μL of virus per well (250μL of guide 1 and 250μL of guide 2 lentivirus were added to each replicate, for region r8 500μL of guide 1 was added). 2 guides per well were used with the exception of region r8 where only one guide was tested due to sequence content of the target region and difficulties designing and cloning site specific guides for this region. pLV hU6-sgRNA hUbC-dCas9-KRAB-T2a-GFP (addgene 71237) was used in a separate well as a control. The next day, media was swapped on the NPCs to media + puromycin (0.5μg/mL) (Gibco A1113803) to select transduced cells. 48hrs after selection, the media on the NPCs to Brain Phys Differentiation media (BrainPhys Neuronal Medium Stemcell Technologies 05790, 1X N2 Supplement-A Stemcell Technologies 07152, 1X NeuroCult SM1 Neuronal Supplement Stemcell Technologies 05711, 10μg human recombinant GDNF Stemcell Technologies, 10μg human recombinant BDNF Stemcell Technologies 78005, 500μL of 200μM L-Ascorbic acid Sigma A0278, 0.5mg Laminin Mouse Protein Gibco 2307015) following the previously published Bardy et al. protocol.^39,53^ After 14 days of differentiation, the cells were collected using the Norgen RNA purification kit (Norgen 17200 and 25710). RNA was sent to Novogene for mRNAseq at 20 million paired end reads per sample on an Illumina NovaSeq X Plus. All mammalian cells were grown at 37°C and 5% CO_2_. pLV hU6-sgRNA hUbC-dCas9-KRAB-T2a-Puro was a gift from Charles Gersbach (Addgene plasmid # 71236; http://n2t.net/addgene:71236; RRID:Addgene_71236).^102^ pLV hU6-sgRNA hUbC-dCas9-KRAB-T2a-GFP was a gift from Charles Gersbach (Addgene plasmid # 71237; http://n2t.net/addgene:71237; RRID:Addgene_71237).^102^ psPAX2 was a gift from Didier Trono (Addgene plasmid # 12260; http://n2t.net/addgene:12260; RRID:Addgene_12260). pMD2.G was a gift from Didier Trono (Addgene plasmid # 12259; http://n2t.net/addgene:12259; RRID:Addgene_12259).

#### RNAseq

Cutadapt^103^ was used to trim adapters from reads before aligning them to GENCODE v42 using STAR aligner.^104^ The reads were indexed using Samtools^105^ and deduplicated using picard^106^. Reads were counted using htseq-count^107^. CPMs were generated using edgeR.^108^ To account for the varying numbers of cell types in the culture, a cell type score was assigned to each replicate using Seurat^54^ and a list of gene markers: neurons (*RBFOX3*, *SYP*), NPCs (*NES*, *PAX6*), and astrocytes (*ALDH1L1*, *SLC1A2*, *SLC1A3*, *S100B*). DESeq2^55^ was used to analyze each target region against a guide targeting the AAV Safe Harbor Locus^109,110^ filtering for a CPM of at least 1 with and without including cell type scores in the model.

### AI tools

#### AlphaGenome MAPT Suturation Mutagenesis

The AlphaGenome API was accessed via the DnaClient using Google Colab.^111^ Hg38v46 annotations were used as reference and a 1 Mb context window around *MAPT* was constructed for predicting variant effects. A saturation mutagenesis window encompassing a window 1kb upstream and downstream of the *MAPT* TSS was built. In silico mutagenesis was then conducted using the recommended RNAseq variant scorer and AlphaGenome’s built in saturation mutagenesis function. The effect of each SNV on RNAseq signal for each gene and pseudogene within the context window was compared against the effect of the reference allele as described by Avsec et al.^68^ resulting in raw scores. Raw scores predicting gene expression changes for *MAPT* in glutamatergic neurons (CL:0000679) were subsequently extracted and exported for analysis in RStudio.

#### PromoterAI MAPT Mutagenesis

A file representing all possible SNVs within 1kb upstream and downstream of the *MAPT* TSS was generated as specified by Jaganathan et al.^69^ Using an input sequence length of 20,480 bp centered around each variant, PromoterAI scored the effect of each SNVs on gene expression using the promoterAI_v1_hg38_mm10_finetune model and GCF_000001405.26 RefSeq. Variant scores were appended to each SNV and imported into RStudio for analysis.

#### Graphing

Plots were made using ggplot2^112^, plotgardener^113^, complexheatmap^114^, or Graphpad prism 10. Graphical figures were made in BioRender and Adobe Illustrator.

#### External Data

Known genetic variants were acquired from the Alzheimer’s Disease Sequencing Project (ADSP) (NIAGADS NG00067). Transcription factor binding and H3K27ac ChIP-seq data was generated by Loupe et al.^64^ and is available through the PsychENCODE Consortium at https://doi.org/10.7303/syn51942384.1. Brain single nucleus multiomics data was generated by Anderson et al.^52^ and is available at GEO accession number GSE214637. *MAPT* glutamatergic neuron HiC, *MAPT* glutamatergic neuron CaptureC, *MAPT* gabaergic cell Capture C, and cultured neuron single nucleus multiomics data were generated by Rogers et al.^39^ and are available at GSE228121. PhyloP conservation scores were generated by the Zoonomia Consortium^71,72^ and obtained from https://hgdownload.cse.ucsc.edu/goldenpath/hg38/cactus241way/. ENCODE cCREs were generated by Moore et al.^51^ and are available on SCREEN (https://screen.wenglab.org).

## Supplementary Material

Supplement 1

Supplement 2

## Data Availability

Processed and raw data generated in this study will be made available through NCBI GEO. All original code generated for this study will be made available at https://github.com/HudsonAlpha/Hauser_MAPT_MPRAs.

## Abbreviations

dELS: distal enhancer like signature
pELS: proximal enhancer like signature
TF: transcription factor signal
CA-CTCF: chromatin accessible, CTCF
CA: chromatin accessible
PLS: promoter like signature
CA-TF: chromatin accessible, transcription factor signature
CA-H3K4me3: chromatin accessible, H3K4me3 signal
MPRA: Massively Parallel Reporter Assay
CRE: Cis-regulatory Element
logFC: log(fold change)
HEKs: HEK293FT cells
AI: artificial intelligence

## References

[1] Courade J.-P., Zetterberg H., Höglinger G.U., and Dewachter I. (2025). The evolving landscape of Alzheimer’s disease therapy: From Aβ to tau. Cell 188, 7337–7354. 10.1016/j.cell.2025.11.033. 41448139

[2] Chong F.P., Ng K.Y., Koh R.Y., and Chye S.M. (2018). Tau Proteins and Tauopathies in Alzheimer’s Disease. Cell Mol Neurobiol 38, 965–980. 10.1007/s10571-017-0574-1. 29299792 PMC11481908

[3] Morris M., Hamto P., Adame A., Devidze N., Masliah E., and Mucke L. (2013). Age-appropriate cognition and subtle dopamine-independent motor deficits in aged Tau knockout mice. Neurobiology of Aging 34, 1523–1529. 10.1016/j.neurobiolaging.2012.12.003. 23332171 PMC3596503

[4] DeVos S.L., Corjuc B.T., Commins C., Dujardin S., Bannon R.N., Corjuc D., Moore B.D., Bennett R.E., Jorfi M., Gonzales J.A., (2018). Tau reduction in the presence of amyloid-β prevents tau pathology and neuronal death in vivo. Brain 141, 2194–2212. 10.1093/brain/awy117. 29733334 PMC6022692

[5] DeVos S.L., Miller R.L., Schoch K.M., Holmes B.B., Kebodeaux C.S., Wegener A.J., Chen G., Shen T., Tran H., Nichols B., (2017). Tau reduction prevents neuronal loss and reverses pathological tau deposition and seeding in mice with tauopathy. Sci Transl Med 9, eaag0481. 10.1126/scitranslmed.aag0481. PMC5792300 28123067

[6] Wegmann S., Maury E.A., Kirk M.J., Saqran L., Roe A., DeVos S.L., Nicholls S., Fan Z., Takeda S., Cagsal-Getkin O., (2015). Removing endogenous tau does not prevent tau propagation yet reduces its neurotoxicity. EMBO J 34, 3028–3041. 10.15252/embj.201592748. 26538322 PMC4687785

[7] Roberson E.D., Scearce-Levie K., Palop J.J., Yan F., Cheng I.H., Wu T., Gerstein H., Yu G.-Q., and Mucke L. (2007). Reducing Endogenous Tau Ameliorates Amyloid β-Induced Deficits in an Alzheimer’s Disease Mouse Model. Science 316, 750–754. 10.1126/science.1141736. 17478722

[8] Voskobiynyk Y., Li Z., Cochran J.N., Davis M.N., Carullo N.V.N., Creed R.B., Buckingham S.C., Hall A.M., Wilson S.M., and Roberson E.D. (2024). Excitoprotective effects of conditional tau reduction in excitatory neurons and in adulthood. bioRxiv, 2024.05.14.594246. 10.1101/2024.05.14.594246.

[9] Congdon E.E., Ji C., Tetlow A.M., Jiang Y., and Sigurdsson E.M. (2023). Tau-targeting therapies for Alzheimer disease: current status and future directions. Nat Rev Neurol 19, 715–736. 10.1038/s41582-023-00883-2. 37875627 PMC10965012

[10] Novak P., Kovacech B., Katina S., Schmidt R., Scheltens P., Kontsekova E., Ropele S., Fialova L., Kramberger M., Paulenka-Ivanovova N., (2021). ADAMANT: a placebo-controlled randomized phase 2 study of AADvac1, an active immunotherapy against pathological tau in Alzheimer’s disease. Nat Aging 1, 521–534. 10.1038/s43587-021-00070-2. 37117834

[11] Höglinger G.U., Litvan I., Mendonca N., Wang D., Zheng H., Rendenbach-Mueller B., Lon H.-K., Jin Z., Fisseha N., Budur K., (2021). Safety and efficacy of tilavonemab in progressive supranuclear palsy: a phase 2, randomised, placebo-controlled trial. The Lancet Neurology 20, 182–192. 10.1016/S1474-4422(20)30489-0. 33609476

[12] Florian H., Wang D., Arnold S.E., Boada M., Guo Q., Jin Z., Zheng H., Fisseha N., Kalluri H.V., Rendenbach-Mueller B., (2023). Tilavonemab in early Alzheimer’s disease: results from a phase 2, randomized, double-blind study. Brain 146, 2275–2284. 10.1093/brain/awad024. 36730056 PMC10232284

[13] Shulman M., Kong J., O’Gorman J., Ratti E., Rajagovindan R., Viollet L., Huang E., Sharma S., Racine A.M., Czerkowicz J., (2023). TANGO: a placebo-controlled randomized phase 2 study of efficacy and safety of the anti-tau monoclonal antibody gosuranemab in early Alzheimer’s disease. Nat Aging 3, 1591–1601. 10.1038/s43587-023-00523-w. 38012285 PMC10724064

[14] Dam T., Boxer A.L., Golbe L.I., Höglinger G.U., Morris H.R., Litvan I., Lang A.E., Corvol J.-C., Aiba I., Grundman M., (2021). Safety and efficacy of anti-tau monoclonal antibody gosuranemab in progressive supranuclear palsy: a phase 2, randomized, placebo-controlled trial. Nat Med 27, 1451–1457. 10.1038/s41591-021-01455-x. 34385707

[15] Monteiro C., Toth B., Brunstein F., Bobbala A., Datta S., Ceniceros R., Sanabria Bohorquez S.M., Anania V.G., Wildsmith K.R., Schauer S.P., (2023). Randomized Phase II Study of the Safety and Efficacy of Semorinemab in Participants With Mild-to-Moderate Alzheimer Disease: Lauriet. Neurology 101, e1391–e1401. 10.1212/WNL.0000000000207663. 37643887 PMC10573141

[16] Teng E., Manser P.T., Pickthorn K., Brunstein F., Blendstrup M., Sanabria Bohorquez S., Wildsmith K.R., Toth B., Dolton M., Ramakrishnan V., (2022). Safety and Efficacy of Semorinemab in Individuals With Prodromal to Mild Alzheimer Disease: A Randomized Clinical Trial. JAMA Neurol 79, 758–767. 10.1001/jamaneurol.2022.1375. 35696185 PMC9194753

[17] Fleisher A.S., Munsie L.M., Perahia D.G.S., Andersen S.W., Higgins I.A., Hauck P.M., Lo A.C., Sims J.R., Brys M., Mintun M., (2024). Assessment of Efficacy and Safety of Zagotenemab: Results From PERISCOPE-ALZ, a Phase 2 Study in Early Symptomatic Alzheimer Disease. Neurology 102, e208061. 10.1212/WNL.0000000000208061. PMC11067698 38386949

[18] Willis B.A., Lo A.C., Dage J.L., Shcherbinin S., Chinchen L., Andersen S.W., LaBell E.S., Perahia D.G.S., Hauck P.M., and Lowe S.L. (2023). Safety, Tolerability, and Pharmacokinetics of Zagotenemab in Participants with Symptomatic Alzheimer’s Disease: A Phase I Clinical Trial. J Alzheimers Dis Rep 7, 1015–1024. 10.3233/ADR-230012. 37849628 PMC10578324

[19] Cai W., Zhang H., Wu Y., Yao Y., and Zhang J. (2024). Comparative the efficacy and safety of Gosuranemab, Semorinemab, Tilavonemab, and Zagotenemab in patients with Alzheimer’s disease: a systematic review and network meta-analysis of randomized controlled trials. Front Aging Neurosci 16, 1465871. 10.3389/fnagi.2024.1465871. PMC11814219 39945003

[20] Kovacech B., Cullen N.C., Novak P., Hanes J., Kontsekova E., Katina S., Parrak V., Fresser M., Vanbrabant J., Feldman H.H., (2024). Post hoc analysis of ADAMANT, a phase 2 clinical trial of active tau immunotherapy with AADvac1 in patients with Alzheimer’s disease, positive for plasma p-tau217. Alzheimers Res Ther 16, 254. 10.1186/s13195-024-01620-7. 39580468 PMC11585249

[21] Mummery C.J., Börjesson-Hanson A., Blackburn D.J., Vijverberg E.G.B., De Deyn P.P., Ducharme S., Jonsson M., Schneider A., Rinne J.O., Ludolph A.C., (2023). Tau-targeting antisense oligonucleotide MAPTRx in mild Alzheimer’s disease: a phase 1b, randomized, placebo-controlled trial. Nat Med 29, 1437–1447. 10.1038/s41591-023-02326-3. 37095250 PMC10287562

[22] Edwards A.L., Collins J.A., Junge C., Kordasiewicz H., Mignon L., Wu S., Li Y., Lin L., DuBois J., Hutchison R.M., (2023). Exploratory Tau Biomarker Results From a Multiple Ascending-Dose Study of BIIB080 in Alzheimer Disease: A Randomized Clinical Trial. JAMA Neurol 80, 1344–1352. 10.1001/jamaneurol.2023.3861. 37902726 PMC10616768

[23] Eli Lilly and Company (2025). A Single- and Multiple-Ascending Dose Study to Evaluate the Safety, Tolerability, Pharmacokinetics, and Pharmacodynamics of LY3954068 in Patients With Early Symptomatic Alzheimer's Disease (clinicaltrials.gov).

[24] Cummings J.L., Zhou Y., Lee G., Zhong K., Fonseca J., Leisgang-Osse A.M., and Cheng F. (2025). Alzheimer’s disease drug development pipeline: 2025. Alzheimer’s & Dementia: Translational Research & Clinical Interventions 11, e70098. 10.1002/trc2.70098. PMC12131090 40463637

[25] Aslanyan A., Foiani M.S., Giovannucci T.A., McDade E., Duff K.E., Mummery C.J., Schöll M., Wildsmith K.R., and Paterson R.W. (2026). Targeting tau in Alzheimer’s Disease: rationale, approach and challenges. Mol Neurodegeneration. 10.1186/s13024-026-00925-5. PMC12973685 41645313

[26] Boettger L.M., Handsaker R.E., Zody M.C., and McCarroll S.A. (2012). Structural haplotypes and recent evolution of the human 17q21.31 region. Nat Genet 44, 881–885. 10.1038/ng.2334. 22751096 PMC4020351

[27] Aranda-Abreu G.E., Rojas-Durán F., Hernández-Aguilar M.E., Herrera-Covarrubias D., García-Hernández L.I., Toledo-Cárdenas M.R., and Chi-Castañeda D. (2025). The Role of Tau in Neuronal Function and Neurodegeneration. Neurology International 17, 75. 10.3390/neurolint17050075. 40423231 PMC12114167

[28] Sadot E., Heicklen-Klein A., Barg J., Lazarovici P., and Ginzburg I. (1996). Identification of a Tau Promoter Region Mediating Tissue-specific-regulated Expression in PC12 Cells. Journal of Molecular Biology 256, 805–812. 10.1006/jmbi.1996.0126. 8601831

[29] Rösler T.W., Tayaranian Marvian A., Brendel M., Nykänen N.-P., Höllerhage M., Schwarz S.C., Hopfner F., Koeglsperger T., Respondek G., Schweyer K., (2019). Four-repeat tauopathies. Progress in Neurobiology 180, 101644. 10.1016/j.pneurobio.2019.101644. 31238088

[30] Drubin D.G., Caput D., and Kirschner M.W. (1984). Studies on the expression of the microtubule-associated protein, tau, during mouse brain development, with newly isolated complementary DNA probes. J Cell Biol 98, 1090–1097. 10.1083/jcb.98.3.1090. 6421824 PMC2113151

[31] Taleghany N., and Oblinger M.M. (1992). Regional distribution and biochemical characteristics of high molecular weight tau in the nervous system. Journal of Neuroscience Research 33, 257–265. 10.1002/jnr.490330209. 1453489

[32] Boyne L.J., Tessler A., Murray M., and Fischer I. (1995). Distribution of big tau in the central nervous system of the adult and developing rat. Journal of Comparative Neurology 358, 279–293. 10.1002/cne.903580209. 7560287

[33] Koppisetti R.K., Barthélemy N.R., Horie K., Ly C.V., Roberts K.F., Koutarapu S., Melendez J., Miller T.M., Sato C., Ghoshal N., (2025). Distribution of big tau isoforms in the human central and peripheral nervous system. bioRxiv, 2025.09.15.676127. 10.1101/2025.09.15.676127. PMC13587115 42473039

[34] Fischer I. (2024). Big tau: What, how, where and why. Cytoskeleton 81, 10–15. 10.1002/cm.21772. 37578198

[35] Fischer I., and Baas P.W. (2020). Resurrecting the Mysteries of Big Tau. Trends Neurosci 43, 493–504. 10.1016/j.tins.2020.04.007. 32434664 PMC7999525

[36] Vacchi E., Kaelin-Lang A., and Melli G. (2020). Tau and Alpha Synuclein Synergistic Effect in Neurodegenerative Diseases: When the Periphery Is the Core. International Journal of Molecular Sciences 21, 5030. 10.3390/ijms21145030. 32708732 PMC7404325

[37] Chung D.-E.C., Deng X., Yalamanchili H.K., Revelli J.-P., Han A.L., Tadros B., Richman R., Dias M., Naini F.A., Boeynaems S., (2024). The big tau splice isoform resists Alzheimer’s-related pathological changes. bioRxiv, 2024.07.30.605685. 10.1101/2024.07.30.605685.

[38] Fischer I., and Baas P.W. (2025). Analyses of exon 4a structure reveal unique properties of Big tau. Preprint at bioRxiv, https://doi.org/10.1101/2025.09.04.674244 10.1101/2025.09.04.674244. PMC12602400 41229558

[39] Rogers B.B., Anderson A.G., Lauzon S.N., Davis M.N., Hauser R.M., Roberts S.C., Rodriguez-Nunez I., Trausch-Lowther K., Barinaga E.A., Hall P.I., (2024). Neuronal MAPT expression is mediated by long-range interactions with cis-regulatory elements. Am J Hum Genet 111, 259–279. 10.1016/j.ajhg.2023.12.015. 38232730 PMC10870142

[40] Bond M.L., Quiroga-Barber I.Y., D’Costa S., Wu Y., Bell J.L., McAfee J.C., Kramer N.E., Lee S., Patrucco M., Phanstiel D.H., (2024). Deciphering the functional impact of Alzheimer’s Disease-associated variants in resting and proinflammatory immune cells. medRxiv, 2024.09.13.24313654. 10.1101/2024.09.13.24313654.

[41] Deng C., Whalen S., Steyert M., Ziffra R., Przytycki P.F., Inoue F., Pereira D.A., Capauto D., Norton S., Vaccarino F.M., (2024). Massively parallel characterization of regulatory elements in the developing human cortex. Science 384, eadh0559. 10.1126/science.adh0559. PMC12085231 38781390

[42] Cooper Y.A., Teyssier N., Dräger N.M., Guo Q., Davis J.E., Sattler S.M., Yang Z., Patel A., Wu S., Kosuri S., (2022). Functional regulatory variants implicate distinct transcriptional networks in dementia. Science 377, eabi8654. 10.1126/science.abi8654. 35981026

[43] Gaynor-Gillett S.C., Cheng L., Shi M., Liu J., Wang G., Spector M., Guo Q., Qi L., Flaherty M., Wall M., (2025). A map of enhancer regions in primary human neural progenitor cells using capture STARR-seq. Genome Res 35, 1887–1901. 10.1101/gr.279584.124. 40645663 PMC12315878

[44] McDiarmid T.A., Page N.F., Chardon F.M., Daza R.M., Chen G.T., Kosicki M., James L.M., Nourie H.C., Laboy-Cintrón D., Lee A.S., (2025). Large-scale discovery of neural enhancers for cis-regulation therapies. bioRxiv, 2025.11.04.686611. 10.1101/2025.11.04.686611.

[45] Lee S., McAfee J.C., Lee J., Gomez A., Ledford A.T., Clarke D., Min H., Gerstein M.B., Boyle A.P., Sullivan P.F., (2025). Massively parallel reporter assay investigates shared genetic variants of eight psychiatric disorders. Cell 188, 1409–1424.e21. 10.1016/j.cell.2024.12.022. 39848247 PMC11890967

[46] Retallick-Townsley K.G., Lee S., Williams S.E., Cartwright S., Cohen S., Sen A., Jia M., Young H., Dobbyn L., Deans M., (2025). Dynamic neuro-immune regulation of psychiatric risk loci in human neurons. Preprint at bioRxiv, https://doi.org/10.1101/2024.07.09.602755 10.1101/2024.07.09.602755.

[47] McAfee J.C., Lee S., Lee J., Bell J.L., Krupa O., Davis J., Insigne K., Bond M.L., Zhao N., Boyle A.P., (2023). Systematic investigation of allelic regulatory activity of schizophrenia-associated common variants. Cell Genomics 3, 100404. 10.1016/j.xgen.2023.100404. PMC10589626 37868037

[48] Anderson A.G., Rogers B.B., Barinaga E.A., Loupe J.M., WaMaina E., Johnston S.Q., Limbo H.L., Gardner E.A., Moyer A.J., Gross A.L., (2025). Single Cell Multiomics Across Nine Mammals Reveals Cell Type Specific Regulatory Conservation in the Brain. Preprint at bioRxiv, https://doi.org/10.1101/2025.08.06.668931 10.1101/2025.08.06.668931.

[49] Pantazis C.B., Yang A., Lara E., McDonough J.A., Blauwendraat C., Peng L., Oguro H., Kanaujiya J., Zou J., Sebesta D., (2022). A reference human induced pluripotent stem cell line for large-scale collaborative studies. Cell Stem Cell 29, 1685–1702.e22. 10.1016/j.stem.2022.11.004. 36459969 PMC9782786

[50] Schmid B., Holst B., Poulsen U., Jørring I., Clausen C., Rasmussen M., Mau-Holzmann U.A., Steeg R., Nuthall H., Ebneth A., (2021). Generation of two gene edited iPSC-lines carrying a DOX-inducible NGN2 expression cassette with and without GFP in the AAVS1 locus. Stem Cell Research 52, 102240. 10.1016/j.scr.2021.102240. 33610017

[51] Moore J.E., Pratt H.E., Fan K., Phalke N., Fisher J., Elhajjajy S.I., Andrews G., Gao M., Shedd N., Fu Y., (2026). An expanded registry of candidate cis-regulatory elements. Nature, 1–10. 10.1038/s41586-025-09909-9. PMC13164889 41501460

[52] Anderson A.G., Rogers B.B., Loupe J.M., Rodriguez-Nunez I., Roberts S.C., White L.M., Brazell J.N., Bunney W.E., Bunney B.G., Watson S.J., (2023). Single nucleus multiomics identifies ZEB1 and MAFB as candidate regulators of Alzheimer’s disease-specific cis-regulatory elements. Cell Genom 3, 100263. 10.1016/j.xgen.2023.100263. PMC10025452 36950385

[53] Bardy C., van den Hurk M., Eames T., Marchand C., Hernandez R.V., Kellogg M., Gorris M., Galet B., Palomares V., Brown J., (2015). Neuronal medium that supports basic synaptic functions and activity of human neurons in vitro. Proc Natl Acad Sci U S A 112, E2725–2734. 10.1073/pnas.1504393112. 25870293 PMC4443325

[54] Hao Y., Stuart T., Kowalski M.H., Choudhary S., Hoffman P., Hartman A., Srivastava A., Molla G., Madad S., Fernandez-Granda C., (2024). Dictionary learning for integrative, multimodal and scalable single-cell analysis. Nat Biotechnol 42, 293–304. 10.1038/s41587-023-01767-y. 37231261 PMC10928517

[55] Love M.I., Huber W., and Anders S. (2014). Moderated estimation of fold change and dispersion for RNA-seq data with DESeq2. Genome Biol 15, 550. 10.1186/s13059-014-0550-8. 25516281 PMC4302049

[56] Fulco C.P., Nasser J., Jones T.R., Munson G., Bergman D.T., Subramanian V., Grossman S.R., Anyoha R., Doughty B.R., Patwardhan T.A., (2019). Activity-by-contact model of enhancer–promoter regulation from thousands of CRISPR perturbations. Nat Genet 51, 1664–1669. 10.1038/s41588-019-0538-0. 31784727 PMC6886585

[57] Pfalzer A.C., Ivers B., Haynam A., Drake B., Koolen D.A., Kasri N.N., de Vries B.B.A., Mefford H.C., Morgan A., Bichell T.J., (2024). Koolen-de Vries Syndrome: a journey from diagnosis to treatments. Ther Adv Rare Dis 5, 26330040241265414. 10.1177/26330040241265414. PMC11287726 39081270

[58] Jun G., Ibrahim-Verbaas C.A., Vronskaya M., Lambert J.-C., Chung J., Naj A.C., Kunkle B.W., Wang L.-S., Bis J.C., Bellenguez C., (2016). A novel Alzheimer disease locus located near the gene encoding tau protein. Mol Psychiatry 21, 108–117. 10.1038/mp.2015.23. 25778476 PMC4573764

[59] Wainberg M., Andrews S.J., and Tripathy S.J. (2023). Shared genetic risk loci between Alzheimer’s disease and related dementias, Parkinson’s disease, and amyotrophic lateral sclerosis. Alzheimers Res Ther 15, 113. 10.1186/s13195-023-01244-3. 37328865 PMC10273745

[60] Ahituv N. (2016). Exonic enhancers: proceed with caution in exome and genome sequencing studies. Genome Med 8, 14. 10.1186/s13073-016-0277-0. 26856702 PMC4745165

[61] Escartin C., Galea E., Lakatos A., O’Callaghan J.P., Petzold G.C., Serrano-Pozo A., Steinhäuser C., Volterra A., Carmignoto G., Agarwal A., (2021). Reactive astrocyte nomenclature, definitions, and future directions. Nat Neurosci 24, 312–325. 10.1038/s41593-020-00783-4. 33589835 PMC8007081

[62] Keukeleire P., Rosen J.D., Göbel-Knapp A., Salomon K., Schubach M., and Kircher M. (2025). Using individual barcodes to increase quantification power of massively parallel reporter assays. BMC Bioinformatics 26, 52. 10.1186/s12859-025-06065-9. 39948460 PMC11827149

[63] Corces M.R., Shcherbina A., Kundu S., Gloudemans M.J., Frésard L., Granja J.M., Louie B.H., Eulalio T., Shams S., Bagdatli S.T., (2020). Single-cell epigenomic analyses implicate candidate causal variants at inherited risk loci for Alzheimer’s and Parkinson’s diseases. Nat Genet 52, 1158–1168. 10.1038/s41588-020-00721-x. 33106633 PMC7606627

[64] Loupe J.M., Anderson A.G., Rizzardi L.F., Rodriguez-Nunez I., Moyers B., Trausch-Lowther K., Jain R., Bunney W.E., Bunney B.G., Cartagena P., (2024). Multiomic profiling of transcription factor binding and function in human brain. Nat Neurosci 27, 1387–1399. 10.1038/s41593-024-01658-8. 38831039 PMC12991031

[65] Wreczycka K., Franke V., Uyar B., Wurmus R., Bulut S., Tursun B., and Akalin A. (2019). HOT or not: examining the basis of high-occupancy target regions. Nucleic Acids Res 47, 5735–5745. 10.1093/nar/gkz460. 31114922 PMC6582337

[66] Anderson A.G., Moyers B.A., Loupe J.M., Rodriguez-Nunez I., Felker S.A., Lawlor J.M.J., Bunney W.E., Bunney B.G., Cartagena P.M., Sequeira A., (2024). Allele-specific transcription factor binding across human brain regions offers mechanistic insight into eQTLs. Genome Res 34, 1224–1234. 10.1101/gr.278601.123. 39152038 PMC11444172

[67] Karczewski K.J., Francioli L.C., Tiao G., Cummings B.B., Alföldi J., Wang Q., Collins R.L., Laricchia K.M., Ganna A., Birnbaum D.P., (2020). The mutational constraint spectrum quantified from variation in 141,456 humans. Nature 581, 434–443. 10.1038/s41586-020-2308-7. 32461654 PMC7334197

[68] Avsec Ž., Latysheva N., Cheng J., Novati G., Taylor K.R., Ward T., Bycroft C., Nicolaisen L., Arvaniti E., Pan J., (2026). Advancing regulatory variant effect prediction with AlphaGenome. Nature 649, 1206–1218. 10.1038/s41586-025-10014-0. 41606153 PMC12851941

[69] Jaganathan K., Ersaro N., Novakovsky G., Wang Y., James T., Schwartzentruber J., Fiziev P., Kassam I., Cao F., Hawe J., (2025). Predicting expression-altering promoter mutations with deep learning. Science 389, eads7373. 10.1126/science.ads7373. 40440429

[70] Jaganathan K., Kyriazopoulou Panagiotopoulou S., McRae J.F., Darbandi S.F., Knowles D., Li Y.I., Kosmicki J.A., Arbelaez J., Cui W., Schwartz G.B., (2019). Predicting Splicing from Primary Sequence with Deep Learning. Cell 176, 535–548.e24. 10.1016/j.cell.2018.12.015. 30661751

[71] Genereux D.P., Serres A., Armstrong J., Johnson J., Marinescu V.D., Murén E., Juan D., Bejerano G., Casewell N.R., Chemnick L.G., (2020). A comparative genomics multitool for scientific discovery and conservation. Nature 587, 240–245. 10.1038/s41586-020-2876-6. 33177664 PMC7759459

[72] Andrews G., Fan K., Pratt H.E., Phalke N., Zoonomia Consortium, Karlsson E.K., Lindblad-Toh K., Gazal S., Moore J.E., and Weng Z. (2023). Mammalian evolution of human cis-regulatory elements and transcription factor binding sites. Science 380, eabn7930. 10.1126/science.abn7930. 37104580

[73] Coetzee S.G., Coetzee G.A., and Hazelett D.J. (2015). motifbreakR: an R/Bioconductor package for predicting variant effects at transcription factor binding sites. Bioinformatics 31, 3847–3849. 10.1093/bioinformatics/btv470. 26272984 PMC4653394

[74] Svaren J., and Meijer D. (2008). The molecular machinery of myelin gene transcription in Schwann cells. Glia 56, 1541–1551. 10.1002/glia.20767. 18803322 PMC2930200

[75] Decker L., Desmarquet-Trin-Dinh C., Taillebourg E., Ghislain J., Vallat J.-M., and Charnay P. (2006). Peripheral Myelin Maintenance Is a Dynamic Process Requiring Constant Krox20 Expression. J. Neurosci. 26, 9771–9779. 10.1523/JNEUROSCI.0716-06.2006. 16988048 PMC6674452

[76] Topilko P., Schneider-Maunoury S., Levi G., Baron-Van Evercooren A., Chennoufi A.B.Y., Seitanidou T., Babinet C., and Charnay P. (1994). Krox-20 controls myelination in the peripheral nervous system. Nature 371, 796–799. 10.1038/371796a0. 7935840

[77] Stolt C.C., and Wegner M. (2016). Schwann cells and their transcriptional network: Evolution of key regulators of peripheral myelination. Brain Research 1641, 101–110. 10.1016/j.brainres.2015.09.025. 26423937

[78] Patel A., Williams M., Hawkins K., Gallo L., Grillo M., Akanda N., Guo X., Lambert S., and Hickman J.J. (2024). Establishment of a Serum-Free Human iPSC-Derived Model of Peripheral Myelination. ACS Biomater Sci Eng 10, 7132–7143. 10.1021/acsbiomaterials.4c01431. 39437333 PMC11558563

[79] Szigeti K., Wiszniewski W., Saifi G.M., Sherman D.L., Sule N., Adesina A.M., Mancias P., Papasozomenos S.C., Miller G., Keppen L., (2007). Functional, histopathologic and natural history study of neuropathy associated with EGR2 mutations. Neurogenetics 8, 257–262. 10.1007/s10048-007-0094-0. 17717711

[80] Chu X.J., Du K., Meng L.C., Xie Z.Y., Zhu Y., Zhang W., Wang Z.X., and Yuan Y. (2022). EGR2-related mixed demyelinating and axonal Charcot-Marie-Tooth disease: An electrodiagnostic, nerve imaging, and histological study. Clin Neuropathol 41, 245–252. 10.5414/NP301460. 35770518

[81] Tozza S., Magri S., Pennisi E.M., Schirinzi E., Pisciotta C., Balistreri F., Severi D., Ricci G., Siciliano G., Taroni F., (2019). A novel family with axonal Charcot-Marie-Tooth disease caused by a mutation in the EGR2 gene. J Peripher Nerv Syst 24, 219–223. 10.1111/jns.12314. 30843326

[82] Poirier R., Cheval H., Mailhes C., Charnay P., Davis S., and Laroche S. (2007). Paradoxical role of an Egr transcription factor family member, Egr2/Krox20, in learning and memory. Front. Behav. Neurosci. 1. 10.3389/neuro.08.006.2007. PMC2525857 18958188

[83] Swanberg S.E., Nagarajan R.P., Peddada S., Yasui D.H., and LaSalle J.M. (2009). Reciprocal co-regulation of EGR2 and MECP2 is disrupted in Rett syndrome and autism. Hum Mol Genet 18, 525–534. 10.1093/hmg/ddn380. 19000991 PMC2638799

[84] Park J.-C., Ryu H., Kim N.-H., Jeon Y.-J., Kang H.J., Choi J.-S., Chung C., and Han J.-S. (2025). Egr2 Modulation in the Medial Prefrontal Cortex Regulates Resistance to Stress-Induced Memory Impairments. Eur Neuropsychopharmacol 96, 9–11. 10.1016/j.euroneuro.2025.03.010. 40220580

[85] Mundackal Sivaraman D., Shaju A., S Nair G., and Balan S. (2026). Systems Biology of Mesial Temporal Lobe Epilepsy and Role of Iron-Related Gene Expression in Its Pathophysiology. OMICS, 15578100261419485. 10.1177/15578100261419485. 41636759

[86] López-López D., Gómez-Nieto R., Herrero-Turrión M.J., García-Cairasco N., Sánchez-Benito D., Ludeña M.D., and López D.E. (2017). Overexpression of the immediate-early genes Egr1, Egr2, and Egr3 in two strains of rodents susceptible to audiogenic seizures. Epilepsy & Behavior 71, 226–237. 10.1016/j.yebeh.2015.12.020. 26775236

[87] Morita K., Okamura T., Sumitomo S., Iwasaki Y., Fujio K., and Yamamoto K. (2016). Emerging roles of Egr2 and Egr3 in the control of systemic autoimmunity. Rheumatology 55, ii76–ii81. 10.1093/rheumatology/kew342. 27856665

[88] Sumitomo S., Fujio K., Okamura T., and Yamamoto K. (2013). Egr2 and Egr3 are the unique regulators for systemic autoimmunity. JAKSTAT 2, e23952. 10.4161/jkst.23952. PMC3710327 24058814

[89] Petazzi P., Jorge-Torres O.C., Gomez A., Scognamiglio I., Serra-Musach J., Merkel A., Grases D., Xiol C., O’Callaghan M., Armstrong J., (2023). Global Impairment of Immediate-Early Genes Expression in Rett Syndrome Models and Patients Linked to Myelination Defects. Int J Mol Sci 24, 1453. 10.3390/ijms24021453. 36674969 PMC9864472

[90] Wallace C.H., Oliveros G., Xie L., Serrano P., Rockwell P., and Figueiredo-Pereira M. (2024). Potential Alzheimer’s early biomarkers in a transgenic rat model and benefits of diazoxide/dibenzoylmethane co-treatment on spatial memory and AD-pathology. Sci Rep 14, 3730. 10.1038/s41598-024-54156-z. 38355687 PMC10867006

[91] Güntan İ., Ghestem A., Nazaruk K., Nizińska K., Olszewski M., Nowicka D., Bernard C., and Łukasiuk K. (2025). Diurnal dynamics of the Zbtb14 protein in the ventral hippocampus are disrupted in epileptic mice. Neuroscience 569, 12–20. 10.1016/j.neuroscience.2025.01.039. 39870297

[92] Roth L.M., Michal M., Michal M., and Cheng L. (2018). Protein expression of the transcription factors DMRT1, TCLF5, and OCT4 in selected germ cell neoplasms of the testis. Human Pathology 82, 68–75. 10.1016/j.humpath.2018.07.019. 30067948

[93] Galán-Martínez J., Berenguer I., del Carmen Maza M., Stamatakis K., Gironès N., and Fresno M. (2022). TCFL5 deficiency impairs the pachytene to diplotene transition during spermatogenesis in the mouse. Sci Rep 12, 10956. 10.1038/s41598-022-15167-w. 35768632 PMC9242989

[94] Samelson A.J., Ariqat N., McKetney J., Rohanitazangi G., Bravo C.P., Bose R.S., Travaglini K.J., Lam V.L., Goodness D., Ta T., (2026). CRISPR screens in iPSC-derived neurons reveal principles of tau proteostasis. Cell 0. 10.1016/j.cell.2025.12.038. PMC12978015 41610849

[95] Kim J., de Haro M., Al-Ramahi I., Garaicoechea L.L., Jeong H.-H., Sonn J.Y., Tadros B., Liu Z., Botas J., and Zoghbi H.Y. (2023). Evolutionarily conserved regulators of tau identify targets for new therapies. Neuron 111, 824–838.e7. 10.1016/j.neuron.2022.12.012. 36610398

[96] Roberts B.S., Partridge E.C., Moyers B.A., Agarwal V., Newberry K.M., Martin B.K., Shendure J., Myers R.M., and Cooper G.M. (2021). Genome-wide strand asymmetry in massively parallel reporter activity favors genic strands. Genome Res 31, 866–876. 10.1101/gr.270751.120. 33879525 PMC8092006

[97] Gordon M.G., Inoue F., Martin B., Schubach M., Agarwal V., Whalen S., Feng S., Zhao J., Ashuach T., Ziffra R., (2020). lentiMPRA and MPRAflow for high-throughput functional characterization of gene regulatory elements. Nat Protoc 15, 2387–2412. 10.1038/s41596-020-0333-5. 32641802 PMC7550205

[98] Cochran J.N., Acosta-Uribe J., Esposito B.T., Madrigal L., Aguillón D., Giraldo M.M., Taylor J.W., Bradley J., Fulton-Howard B., Andrews S.J., (2023). Genetic associations with age at dementia onset in the PSEN1 E280A Colombian kindred. Alzheimers Dement 19, 3835–3847. 10.1002/alz.13021. 36951251 PMC10514237

[99] Moyers B.A., Partridge E.C., Mackiewicz M., Betti M.J., Darji R., Meadows S.K., Newberry K.M., Brandsmeier L.A., Wold B.J., Mendenhall E.M., (2023). Characterization of human transcription factor function and patterns of gene regulation in HepG2 cells. Genome Res 33, 1879–1892. 10.1101/gr.278205.123. 37852782 PMC10760452

[100] Ashuach T., Fischer D.S., Kreimer A., Ahituv N., Theis F.J., and Yosef N. (2019). MPRAnalyze: statistical framework for massively parallel reporter assays. Genome Biol 20, 183. 10.1186/s13059-019-1787-z. 31477158 PMC6717970

[101] Rosen J.D., Vasanthakumari A.D., Salomon K., Lange N. de, Dash P.M., Keukeleire P., Hassan A., Barrera A., Kircher M., Love M.I., (2025). Uniform processing and analysis of IGVF massively parallel reporter assay data with MPRAsnakeflow. Preprint at bioRxiv, https://doi.org/10.1101/2025.09.25.678548 10.1101/2025.09.25.678548. PMC13534247 42581006

[102] Thakore P.I., D’Ippolito A.M., Song L., Safi A., Shivakumar N.K., Kabadi A.M., Reddy T.E., Crawford G.E., and Gersbach C.A. (2015). Highly specific epigenome editing by CRISPR-Cas9 repressors for silencing of distal regulatory elements. Nat Methods 12, 1143–1149. 10.1038/nmeth.3630. 26501517 PMC4666778

[103] Martin M. (2011). Cutadapt removes adapter sequences from high-throughput sequencing reads. EMBnet.journal 17, 10–12. 10.14806/ej.17.1.200.

[104] Dobin A., Davis C.A., Schlesinger F., Drenkow J., Zaleski C., Jha S., Batut P., Chaisson M., and Gingeras T.R. (2013). STAR: ultrafast universal RNA-seq aligner. Bioinformatics 29, 15–21. 10.1093/bioinformatics/bts635. 23104886 PMC3530905

[105] Li H., Handsaker B., Wysoker A., Fennell T., Ruan J., Homer N., Marth G., Abecasis G., Durbin R., and 1000 Genome Project Data Processing Subgroup (2009). The Sequence Alignment/Map format and SAMtools. Bioinformatics 25, 2078–2079. 10.1093/bioinformatics/btp352. 19505943 PMC2723002

[106] Picard toolkit (2019). Broad Institute, GitHub repository.

[107] Anders S., Pyl P.T., and Huber W. (2015). HTSeq—a Python framework to work with high-throughput sequencing data. Bioinformatics 31, 166–169. 10.1093/bioinformatics/btu638. 25260700 PMC4287950

[108] Chen Y., Chen L., Lun A.T.L., Baldoni P.L., and Smyth G.K. (2025). edgeR v4: powerful differential analysis of sequencing data with expanded functionality and improved support for small counts and larger datasets. Nucleic Acids Res 53, gkaf018. 10.1093/nar/gkaf018. PMC11754124 39844453

[109] Hayashi H., Kubo Y., Izumida M., and Matsuyama T. (2020). Efficient viral delivery of Cas9 into human safe harbor. Sci Rep 10, 21474. 10.1038/s41598-020-78450-8. 33293588 PMC7722726

[110] Smith J.R., Maguire S., Davis L.A., Alexander M., Yang F., Chandran S., ffrench-Constant C., and Pedersen R.A. (2008). Robust, Persistent Transgene Expression in Human Embryonic Stem Cells Is Achieved with AAVS1-Targeted Integration. Stem Cells 26, 496–504. 10.1634/stemcells.2007-0039. 18024421

[111] Bisong E. (2019). Google Colaboratory. In Building Machine Learning and Deep Learning Models on Google Cloud Platform: A Comprehensive Guide for Beginners, Bisong E., ed. (Apress), pp. 59–64. 10.1007/978-1-4842-4470-8_7.

[112] Wickham H. (2009). ggplot2: Elegant Graphics for Data Analysis (Springer) 10.1007/978-0-387-98141-3.

[113] Kramer N.E., Davis E.S., Wenger C.D., Deoudes E.M., Parker S.M., Love M.I., and Phanstiel D.H. (2022). Plotgardener: cultivating precise multi-panel figures in R. Bioinformatics 38, 2042–2045. 10.1093/bioinformatics/btac057. 35134826 PMC8963281

[114] Gu Z. (2022). Complex heatmap visualization. Imeta 1, e43. 10.1002/imt2.43. 38868715 PMC10989952

