# Supplementary material for: Promoter mutagenesis and a massively parallel reporter screen of the MAPT locus identifies cis-regulatory elements and genetic variation effects
Source: bioRxiv. 2026 Mar 9:2026.03.06.710116. Preprint. [Version 1] doi: 10.64898/2026.03.06.710116 (PMC13060879; doi:10.64898/2026.03.06.710116)

bioRxiv preprint doi: https://doi.org/10.64898/2026.03.06.710116; this version posted March 9, 2026. The copyright holder for this preprint (which was not certified by peer review) is the author/funder, who has granted bioRxiv a license to display the preprint in perpetuity. It is made available under aCC-BY 4.0 International license.

Figure s1

a. Gene track showing alignment of the 24 BACs chosen for the BAC MPRA to hg38. **b**. Bar plot showing the total number of bins with a pval.mad ≤ 0.05 in the BAC MPRA for Neurons and HEKs, as well as a reduced count where consecutive bins were merged into a single active region. **c.** Bar plot showing the total number of oligos with a pval.mad ≤ 0.05 in the Oligo MPRA, as well as a reduced count where consecutive and overlapping oligos were merged into a single active region.

Figure s2

a–f. Gene tracks of CREs for *MAPT* identified in Rogers et al.[39] where variant effects were found in our MPRA. Regions are labeled as in Rogers et al.[39] as distance from the *MAPT* promoter. Variant effects shown are in Neurons. Adj. p. values for variants are: **a.** chr17:45219428:C:T = 0.0456, chr17:45219449:T:A = 0.0401, **b.** chr17:45241205:C:T = 0.0467, chr17:45241344:C:G = 0.0246, chr17:45241498:G:A = $8.16x10^{-4}$, chr17:45241588:C:T = $3.74x10^{-4}$, chr17:45241965:C:T = < $2.2x10^{-16}$, chr17:45242096:C:T = 0.0246, chr17:45242183:G:GA = 0.0165, **c.** chr17:45429574:G:A = 0.0175, chr17:45429600:C:T = $5.82x10^{-4}$, chr17:45429596:T:C = $3.17x10^{-13}$, chr17:45429616:G:T = $2.82x10^{-5}$, chr17:45429635:C:T = $2.04x10^{-3}$, chr17:45429804:T:G = $2.64x10^{-3}$, **d.** chr17:45431618:C:G = 0.0222, chr17:45431806:C:T = $1.17x10^{-4}$, chr17:45432539:C:T = 0.0476, chr17:45432576:C:T = $7.80x10^{-3}$, chr17:45432583:C:T = 0.0255, chr17:45432606:G:T = < $2.2x10^{-16}$, chr17:45432617:G:T = $1.88x10^{-4}$, chr17:45432652:A:ACCCTT = $4.32x10^{-3}$, **e.** chr17:45848799:G:GC = 0.0312, chr17:45849095:A:G = $1.88x10^{-4}$, chr17:45849813:C:T = $5.08x10^{-3}$, **f.** chr17:45873854:T:G = $7.38x10^{-3}$, chr17:45873875:G:A = 0.0237.

Figure s3

a. Gene track of the *MAPT* promoter saturation mutagenesis region showing variant effects observed in HEK293FT cells (pval.mad ≤ 0.05 and |logFC| > 0.1). The top row of the heatmap represents the centered 5 bp deletion sequences, and the bottom 3 rows represent each alternate base at that position. Red lines represent variants with a gain of activity and blue lines represent a loss of activity. **b.** Correlation plot of logFC of variant effects observed in Neurons and HEK293FT cells (pval.mad ≤ 0.05 and a |logFC| of at least 0.1). Blue dots represent variant effects in HEK293FT cells, pink represent variant effects in Neurons, and purple are variant effects in both cell types. (logFC Spearman's ρ = 0.336, approximate p < $2.2x10^{-16}$)

Figure s4

a. Correlation plots of AlphaGenome raw scores and MPRA logFC from the *MAPT* promoter mutagenesis. (Spearman's ρ = 0.0277, approximate p = 0.0321) **b.** Same as a. except only variants with an adj. p. value ≤ 0.05 in the Neuron MPRA by bcalm are plotted. (Spearman's ρ = $8.71x10^{-9}$, approximate p = $8.71x10^{-4}$) **c.** Correlation plots of PromoterAI scores and MPRA logFC from the *MAPT* promoter mutagenesis. (Spearman's ρ = 0.482, approximate p = $1.89x10^{-4}$) **d.** Same as in c except only variants with an adj. p. value ≤ 0.05 in the Neuron MPRA by bcalm are plotted. (Spearman's ρ = 0.2924, approximate p = $1.10x10^{-9}$) **e.** Correlation plot of

bioRxiv preprint doi: https://doi.org/10.64898/2026.03.06.710116; this version posted March 9, 2026. The copyright holder for this preprint (which was not certified by peer review) is the author/funder, who has granted bioRxiv a license to display the preprint in perpetuity. It is made available under aCC-BY 4.0 International license.

AlphaGenome raw scores and PromoterAI scores for each SNV in the saturation mutagenesis MPRA (Spearman's ρ = 0.153, approximate $p < 2.2\times10^{-16}$). For all plots, the best fit line is a linear regression of the data.

Figure s1

bioRxiv preprint doi: https://doi.org/10.64898/2026.03.06.710116; this version posted March 9, 2026. The copyright holder for this preprint (which was not certified by peer review) is the author/funder, who has granted bioRxiv a license to display the preprint in perpetuity. It is made available under aCC-BY 4.0 International license.

a.

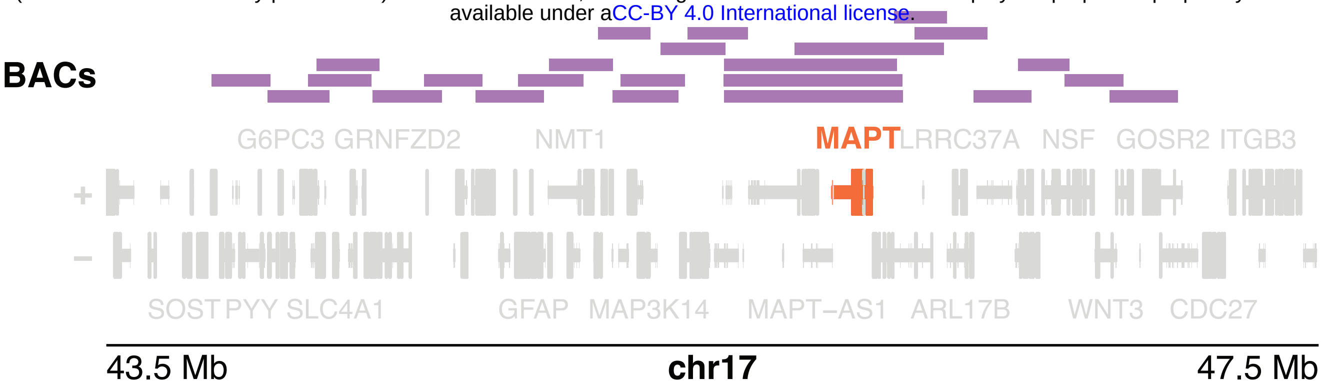

b.

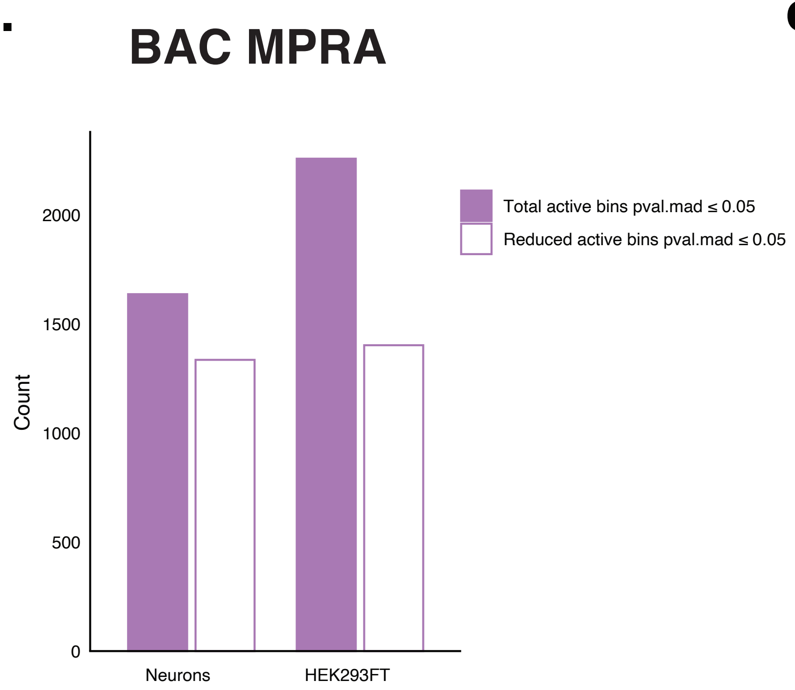

c.

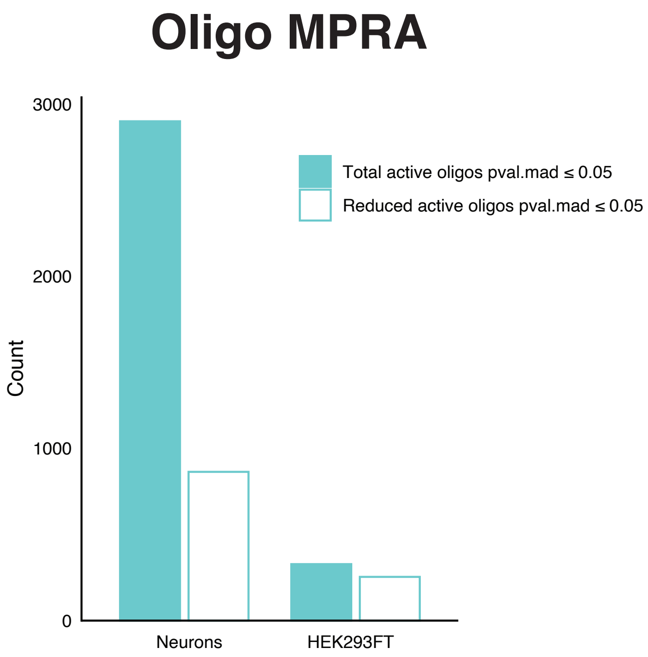

Figure s2

bioRxiv preprint doi: https://doi.org/10.64898/2026.03.06.710116; this version posted March 9, 2026. The copyright holder for this preprint (which was not certified by peer review) is the author/funder, who has granted bioRxiv a license to display the preprint in perpetuity. It is made available under aCC-BY 4.0 International license.

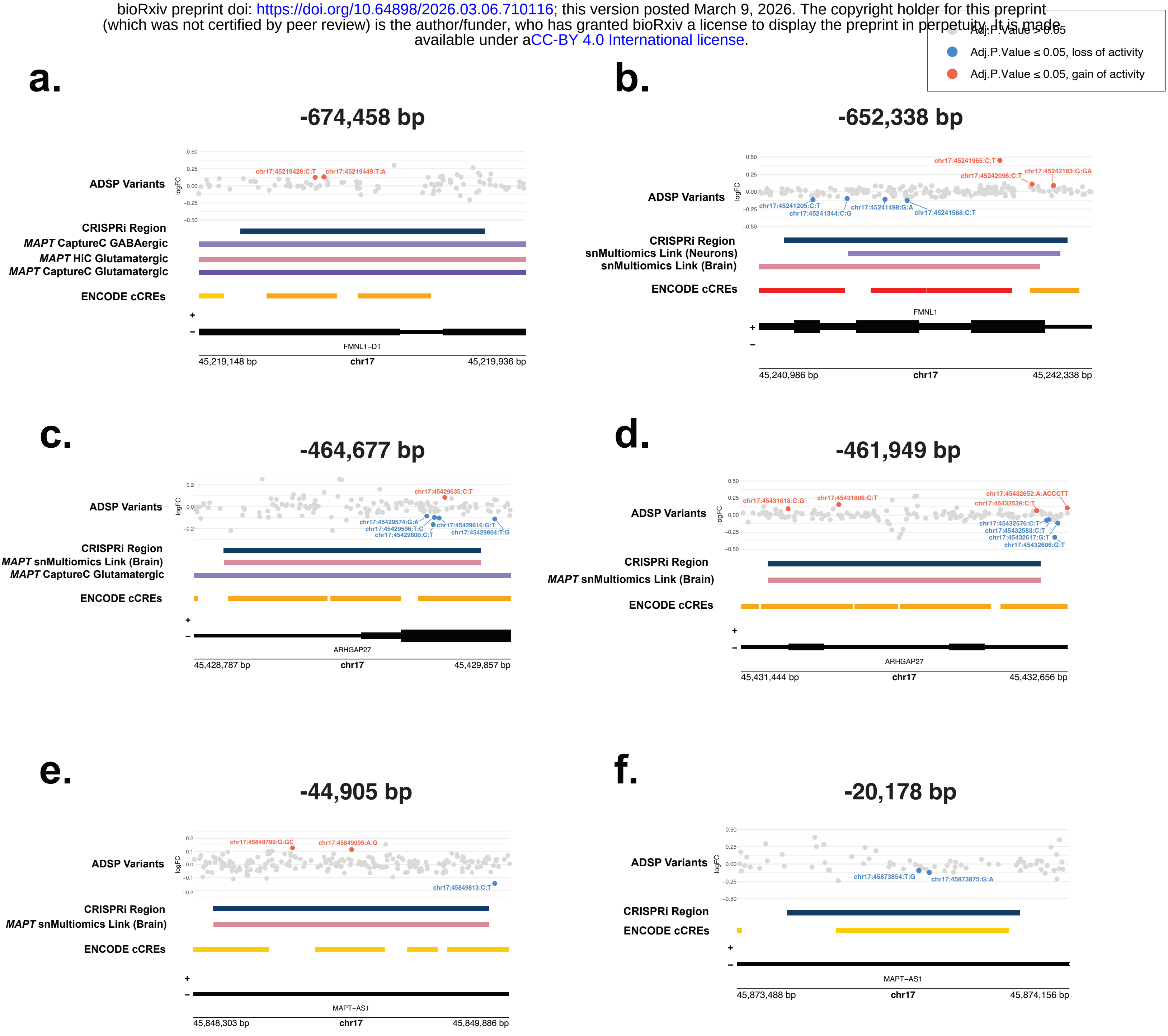

Figure s3

bioRxiv preprint doi: https://doi.org/10.64898/2026.03.06.710116; this version posted March 9, 2026. The copyright holder for this preprint (which was not certified by peer review) is the author/funder, who has granted bioRxiv a license to display the preprint in perpetuity. It is made available under aCC-BY 4.0 International license.

a.

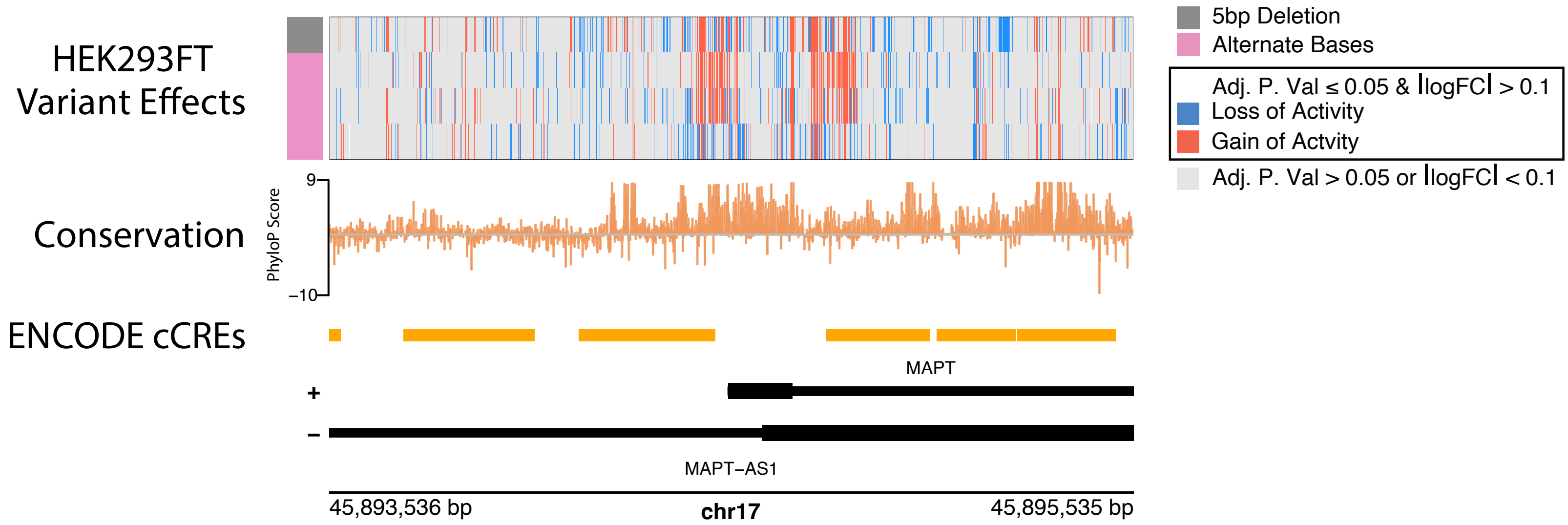

b.

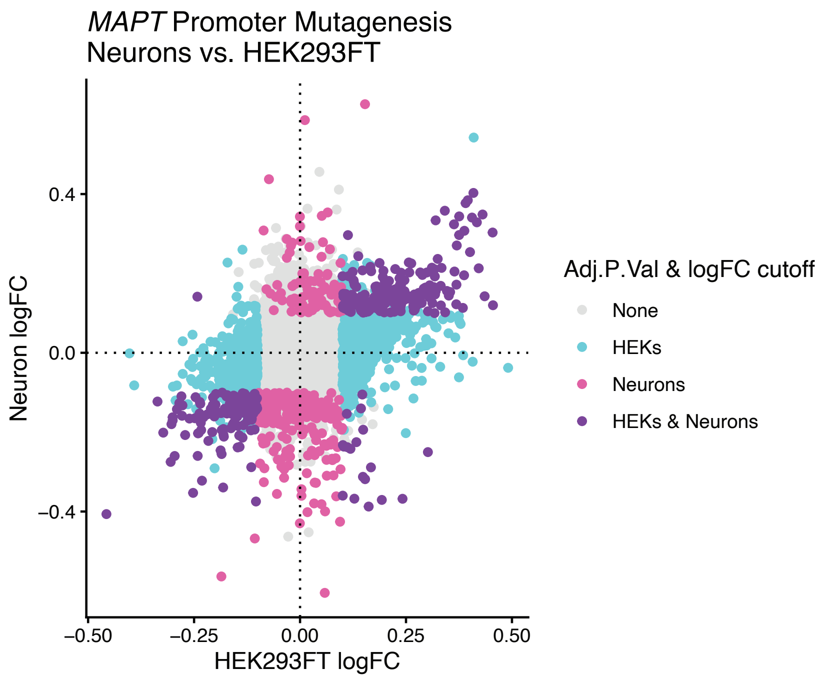

Figure s4

bioRxiv preprint doi: https://doi.org/10.64898/2026.03.06.710116; this version posted March 9, 2026. The copyright holder for this preprint (which was not certified by peer review) is the author/funder, who has granted bioRxiv a license to display the preprint in perpetuity. It is made available under aCC-BY 4.0 International license.

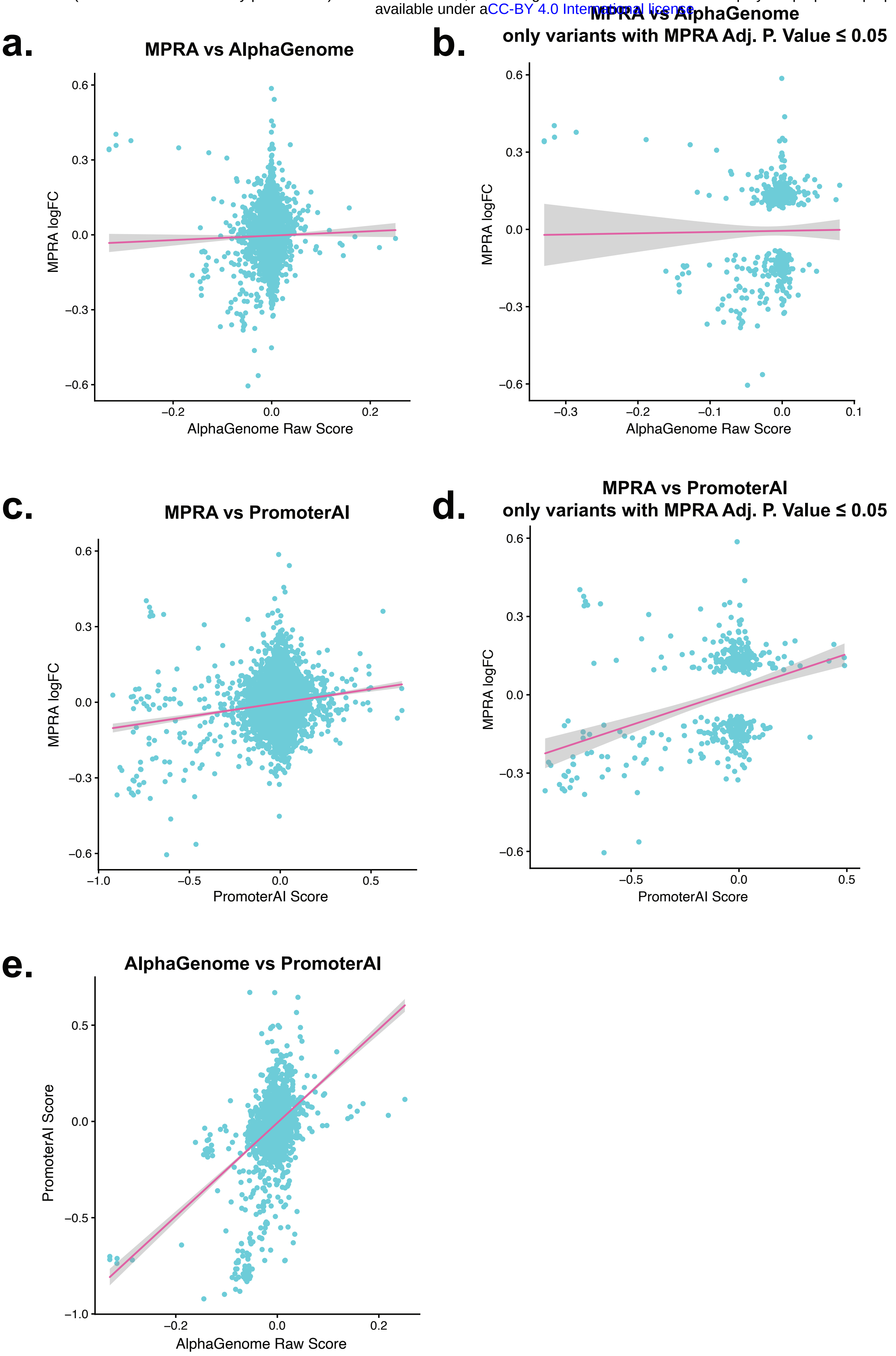

Supplement: Supplement 2 [file NIHPP2026.03.06.710116v1-supplement-2.pdf]
